# Removal of senescent cells reduces the viral load and attenuates pulmonary and systemic inflammation in SARS-CoV-2-infected, aged hamsters

**DOI:** 10.1038/s43587-023-00442-w

**Published:** 2023-07-06

**Authors:** Lou Delval, Aline Hantute-Ghesquier, Valentin Sencio, Jean Michel Flaman, Cyril Robil, Fabiola Silva Angulo, Larissa Lipskaia, Ozmen Çobanoğlu, Anne-Sophie Lacoste, Arnaud Machelart, Adeline Danneels, Mathieu Corbin, Lucie Deruyter, Séverine Heumel, Thierry Idziorek, Karin Séron, Florent Sauve, Antonino Bongiovanni, Vincent Prévot, Isabelle Wolowczuk, Sandrine Belouzard, Jean-Michel Saliou, Philippe Gosset, David Bernard, Yves Rouillé, Serge Adnot, Martine Duterque-Coquillaud, François Trottein

**Affiliations:** 1 Université de Lille, CNRS, INSERM, CHU Lille, Institut Pasteur de Lille, U1019-UMR 9017, Center for Infection and Immunity of Lille, Lille, France; 2 Université de Lille, CNRS, INSERM, CHU Lille, UMR9020-U1277, Institut Pasteur de Lille-CANTHER, Lille, France; 3 Université de Lyon, CNRS, INSERM, U1052-UMR 5286, Centre de Recherche en Cancérologie de Lyon, Centre Léon Bérard, Lyon, France; 4 Université de Paris-Est Créteil, INSERM U955, Institut Mondor de Recherche Biomédicale, Créteil, France; 5 Université de Lille, CNRS, INSERM, CHU Lille, Institut Pasteur de Lille, US 41-UAR 2014, Platforms Lille in Biology & Health, Lille, France; 6 Université de Lille, INSERM, CHU Lille, U1172-UMR 9017, Lille Neuroscience & Cognition Research Center, Lille, France

**Keywords:** SARS-CoV-2, Diseases

## Abstract

Older age is one of the strongest risk factors for severe COVID-19. In this study, we determined whether age-associated cellular senescence contributes to the severity of experimental COVID-19. Aged golden hamsters accumulate senescent cells in the lungs, and the senolytic drug ABT-263, a BCL-2 inhibitor, depletes these cells at baseline and during SARS-CoV-2 infection. Relative to young hamsters, aged hamsters had a greater viral load during the acute phase of infection and displayed higher levels of sequelae during the post-acute phase. Early treatment with ABT-263 lowered pulmonary viral load in aged (but not young) animals, an effect associated with lower expression of ACE2, the receptor for SARS-CoV-2. ABT-263 treatment also led to lower pulmonary and systemic levels of senescence-associated secretory phenotype factors and to amelioration of early and late lung disease. These data demonstrate the causative role of age-associated pre-existing senescent cells on COVID-19 severity and have clear clinical relevance.

## Abstract (web summary)

Delval, Hantute-Ghesquier, Sencio and colleagues demonstrate that depletion of age-associated senescent cells decreases pulmonary viral load and ameliorates early and late lung COVID-19 in a hamster model of aging.

## Main

Older adults are particularly susceptible to respiratory viral infections, and infection with SARS-CoV-2 is no exception^1–5^. This greater susceptibility is mainly related to impairments in pulmonary functions and immune responses in the aging lung^5–7^. Immunosenescence and inflammaging are key features of the aging immune system, in which an accumulation of senescent cells participates in this decline and favors an inflammatory phenotype^8–12^. These changes have a critical role in disease progression and clinical outcomes thereafter. Cellular senescence is a response to stress that stably alters cell functions, including the ability to divide and replicate, and triggers resistance to apoptosis. The latter is due, at least in part, to members of the B cell lymphoma-2 family of proteins (BCL-2, BCL-XL and BCL-W)^13^. Cellular senescence can be triggered by short telomeres or by various stress stimuli, such as DNA-damaging agents, oxidative stress, inflammation and infection^10^. The lungs are particularly exposed to stress stimuli. As a consequence, both naturally occurring senescent cells (in a programmed response) and stress-induced senescent cells accumulate in the aged lung tissue and contribute substantially to the decline in pulmonary functions^14–16^.

Although senescence is beneficial in tumor suppression and wound healing^17–19^, the aberrant accumulation of senescent cells can generate an inflammatory milieu that leads to chronic tissue damage and diseases^10,13^. The senescent cells’ detrimental role mostly concerns the release of various effectors, including inflammatory cytokines, immune modulators, proteases, growth factors, profibrotic factors and pro-coagulant factors that can alter tissue microenvironments^13,20^. This senescence-associated secretory phenotype (SASP) contributes to acute and chronic inflammation and might have a role in immune response regulation. It has been shown that the genetic or pharmacological removal of senescent cells ameliorates disease outcomes and prolongs the lifespan^16,21–25^. Senolytics constitute an emerging class of drugs that can selectively kill senescent cells by exploiting the latter’s differences with non-senescent cells. They may be a tractable treatment option for humans in many settings, ranging from chronic inflammatory diseases to infections^16^. The most promising senolytics to date target anti-apoptosis pathways, which are upregulated in senescent cells. Several such drug candidates, including ABT-263 (navitoclax), target BCL-2 family proteins^22,26^.

The putative role of pre-existing senescent cells in COVID-19 in older patients, as well as in patients with chronic disease conditions, has been suggested^12,27–30^, but experimental evidence is still lacking. Camell et al.^31^ recently investigated the role of age-dependent, pre-existing senescent cells during an infection with a hepatotropic and neurotropic betacoronavirus (mouse hepatitis virus) in aged mice. Treatment with a senolytic or the genetic ablation of senescent cells reduced the viral load in feces, lowered the level of liver and gut inflammation and increased the survival rate. However, it was not clear whether the data on the mouse hepatitis virus can be extrapolated to SARS-CoV-2. Indeed, the mechanism involved in viral entry into cells and the pathogenesis are very different between mouse hepatitis virus and SARS-CoV-2 (ref. ^32^). To address this question, we studied the well-characterized golden hamster (*Mesocricetus auratus*) model^33,34^. Using aged hamsters (22 months old), we first demonstrated that senescent cells, mostly epithelial cells, accumulate in lungs. We analyzed the outcome of a SARS-CoV-2 infection in aged hamsters with regard to the viral load and disease features in the lungs and verified that senescent cells are depleted by treatment with ABT-263. We next analyzed the effect of ABT-263 in this model of COVID-19. Removal of senescent cells in aged hamsters reduced the viral load in lungs, diminished the SASP response in lungs and blood and dampened early and long-term pulmonary disease features upon challenge with SARS-CoV-2. The lower viral replication due to senescent cell depletion associated with a reduced expression of angiotensin-converting enzyme 2 (ACE2), the receptor for SARS-CoV-2. It is noteworthy that we observed age-specific differences in the action of ABT-263 in hamsters, with a lack of effect in young animals. We conclude that the depletion of pre-existing (aged individuals) senescent cells might ameliorate acute and longer-term COVID-19 outcomes.

## Results

### ABT-263 depletes senescent cells in hamster aged lungs

**Fig. 1:**
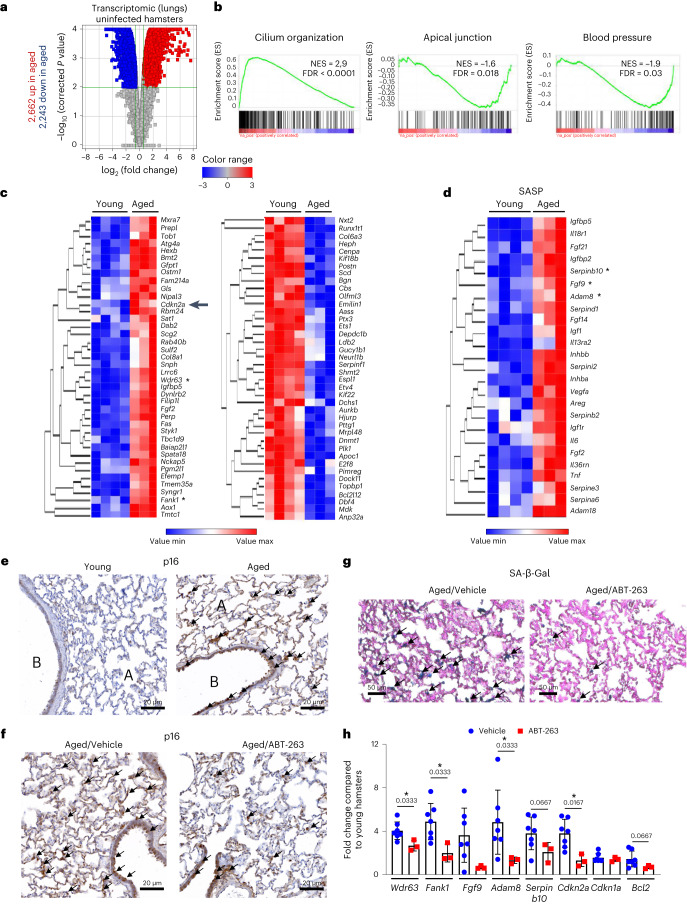
The transcriptomic analysis of aged lungs and the effect of ABT-263 treatment on senescent cells in the lungs. **a**–**d**, Transcriptomic data were generated from whole lung tissue collected from naive aged hamsters (22 months of age) and naive young hamsters (2 months of age) (*n* = 3 and *n* = 4, respectively). Significant differentially expressed genes in aged lungs with fold change cutoffs >1.5 or <1.5 and moderated *t*-test *P* < 0.01 after Benjamani–Hochberg correction were considered. **a**, Volcano plot of transcriptomic data is depicted. The *x* axis represents the log_2_-transformed fold change for differentially expressed genes in aged versus young lungs, and the *y* axis represents the log_10_. **b**, GSEA plots showing the enrichment of upregulated genes (red) and repressed genes (blue) in aged hamsters relative to young hamsters at baseline. The *y* axis indicates the normalized enrichment score (NES). **c**,**d**, Heat maps showing significantly modulated genes related to ‘cellular senescence’ (**c**) and SASP-related factors (**d**) with the highest fold change in expression. The log_2_ expression range values are indicated by the color scale. Asterisks denote genes shown in **h**. **e**, Lungs from aged hamsters and young hamsters were stained with anti-p16. Arrows indicate p16-positive cells. A, lung alveoli; B, bronchi. **f**, The same procedure was repeated but this time in aged hamsters receiving (or not) ABT-263. Scale bars, 20 μm. **g**, SA-β-Gal staining of lung sections after ABT-263 treatment in aged hamsters. **h**, Relative expression levels of transcripts identified in **b** and **c** (marked with an asterisk) and transcripts moderately upregulated in aged lungs but relevant in senescence (*Cdkn1a* and *Bcl2*) as assessed by RT–PCR assays (lung) (*n* = 3–7). The data are expressed as the mean ± s.d. fold change relative to average gene expression in young animals. **a**–**d**, One experiment performed. **e**,**h**, One representative experiment out of two is shown. **h**, Significant differences were determined using the two-tailed Mann–Whitney *U-*test. *P < 0.05.

**Extended Data Fig. 1:**
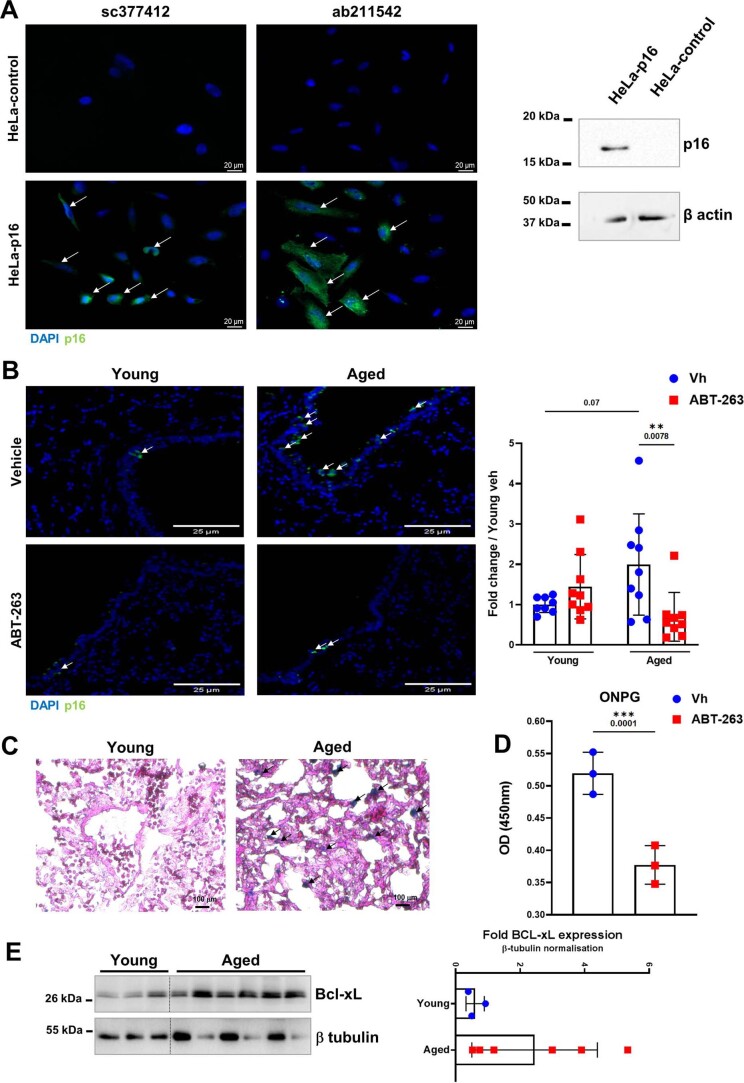
Enhanced cellular senescence in aged lungs and effects of ABT-263 treatment. **A**, Validation of the anti-p16 antibodies on transfected cells. HeLa cells were transfected with the control plasmid pcDNA3.1(+) or the plasmid pcDNA3.1(+) encoding hamster p16. Left panel, Cells were labeled with two different anti-p16 antibodies (immunofluorescence). Right panel, Expression of p16 and β actin in HeLa cells expressing or not hamster p16 as assessed by western blotting. **B**, Left panel, Effect of ABT-263 treatment on the number of p16-positive cells (white arrows) in young and aged lungs as assessed by immunofluorescence. Bars: 25 μm. Right panel, Quantification of p16-expressing cells. The histograms indicate the fold change relative to average intensity in vehicle-treated young animals (n = 8-9). **C**, SA-β-Gal staining of lung sections from young hamsters and aged hamsters. Increased blue staining (black arrows) indicates a higher number of senescent cells in aged lungs**. D**, ONPG-based β-Gal activity of lung extracts collected from aged hamsters treated with the vehicle or with ABT-263 (n = 3). **E**, Left panel, Expression of Bcl-xL and β tubulin in young and aged whole-lung homogenates as assessed by western blotting. Right panel, the relative protein levels normalized to β tubulin are shown (n = 3-6). For all graphs, errors indicate mean ± s.d. Pooled results from two experiments (B) and one of two representative experiments (A, C-E) are shown. Significant differences were determined using the two tailed Mann Whitney *U* test (**D** and **E**) and One-way ANOVA Kruskal-Wallis test (nonparametric), followed by the Dunn’s posttest test (B, right). ** *P* < 0.01, *** *P* < 0.001. Source data

Before investigating the effect of a SARS-CoV-2 infection in aged hamsters, we first analyzed the steady-state level of cellular senescence (bulk transcriptomic on lung samples). Compared with young hamsters (2 months old), a functional characterization of differentially expressed genes by gene set enrichment analysis (GSEA) highlighted changes in several classes of genes in aged (22 months old) hamsters; some genes were upregulated (for example, cilium organization and movement), and others were downregulated (for example, apical junctions and blood pressure) (Fig. 1a,b and Table 1). The CellAge database was then interrogated to identify genes related to cell senescence. We identified 62 upregulated and 102 downregulated senescence-associated genes in aged hamsters (fold change > 1.5, *P* < 0.01). The genes related to cell senescence with the greatest fold changes in expression (upregulation or downregulation) are shown in Fig. 1c. These included the prototypical senescent marker p16^INK4a^ (encoded by*Cdkn2a*, upregulated). With regard to genes encoding SASP-related factors, we found that many cytokines, cytokine receptors, proteases, protease inhibitors (such as serpin) and growth factors were upregulated in the lungs of aged animals, relative to young animals (Fig. 1d). Accumulation of p16-positive senescent cells during aging is critical in multi-organ age-related phenotype^25,35,36^. Thus, p16 expression in aged hamsters was assessed by immunohistochemistry and immunofluorescence on lung sections. The specificity of the anti-p16 was verified using HeLa cells expressing hamster p16 (Extended Data Fig. 1a). The analyses revealed that a large number of cells (mostly bronchial and alveolar epithelial cells) in lungs from aged animals expressed p16 (Fig. 1e and Extended Data Fig. 1b, top). Few p16-positive cells were detected in lungs from young animals. Although senescence-associated β-galactosidase (SA-β-Gal) activity cannot be attributed uniquely to cell senescence^37^, it is considered a good marker of cellular senescence^38–40^. The frequency of SA-β-Gal-positive cells was by far higher in aged lungs relative to young lungs (Extended Data Fig. 1c). Senescent cells express high levels of anti-apoptotic BCL-2 family members, including BCL-XL^13^. In line, pulmonary expression of BCL-XL protein was clearly higher in aged lungs compared to young lungs, as assessed by western blotting (Extended Data Fig. 1d).

**Table 1:** GSEA analysis (lungs from uninfected young hamsters versus uninfected, aged hamsters)

GSEA name	MSigDB^a^	NES^b^	FDR *q* value	Direction^c^
GOBP_CILIUM_ORGANIZATION AND MOVEMENT	C5BP	2.96	<0.00001	Up
REACTOME_GPCR_LIGAND_BINDING	C2CP	−2.28	<0.00001	Down
REACTOME_ECM_PROTEOGLYCANS	C2CP	−2.1	0.0006	Down
GOBP_REGULATION_OF_BLOOD_PRESSURE	C5BP	−1.93	0.03	Down
GOBP_NEGATIVE_REGULATION_OF_LEUKOCYTE_APOPTOTIS	C5BP	−1.9	0.03	Down
HALLMARK_EPITHELIAL_MESENCHYMAL_TRANSITION	Hallmarks	−1.6	0.025	Down
HALLMARK_APICAL_JUNCTION	Hallmarks	−1.59	0.018	Down

^a^Name of the GSEA Molecular Signatures Database (MSigDB) used.

^b^Normalized enrichment score (NES) calculated by GSEA software.

^c^Type of list used for GSEA analysis: upregulated genes (up) or downregulated genes (down) in aged lungs versus young lungs.

The BCL-2 family inhibitor ABT-263 is a potent, selective eliminator of senescent cells in the mouse^22^. The compound’s effect in aged hamsters had not previously been evaluated. ABT-263 treatment for three consecutive days ablated, albeit not completely, p16-expressing cells in hamster lung tissue as revealed by immunohistochemistry and immunofluorescence (Fig. 1f and Extended Data Fig. 1b). Analysis of SA-β-Gal activity confirmed the efficacy of ABT-263 (Fig. 1g and Extended Data Fig. 1e). Accordingly, ABT-263 treatment was associated with strong downregulation of transcripts that encode senescence-associated factors identified as upregulated in aged hamsters (Fig. 1h). ABT-263 also reduced, albeit not significantly, *Bcl2* transcript expression but not *p21* (also known as *Cdkn1a*, another marker of senescence). Aged hamster might constitute a good model for studying the impact of senolysis in COVID-19.

### Aged hamsters develop a high viral load and post-acute sequelae

**Fig. 2:**
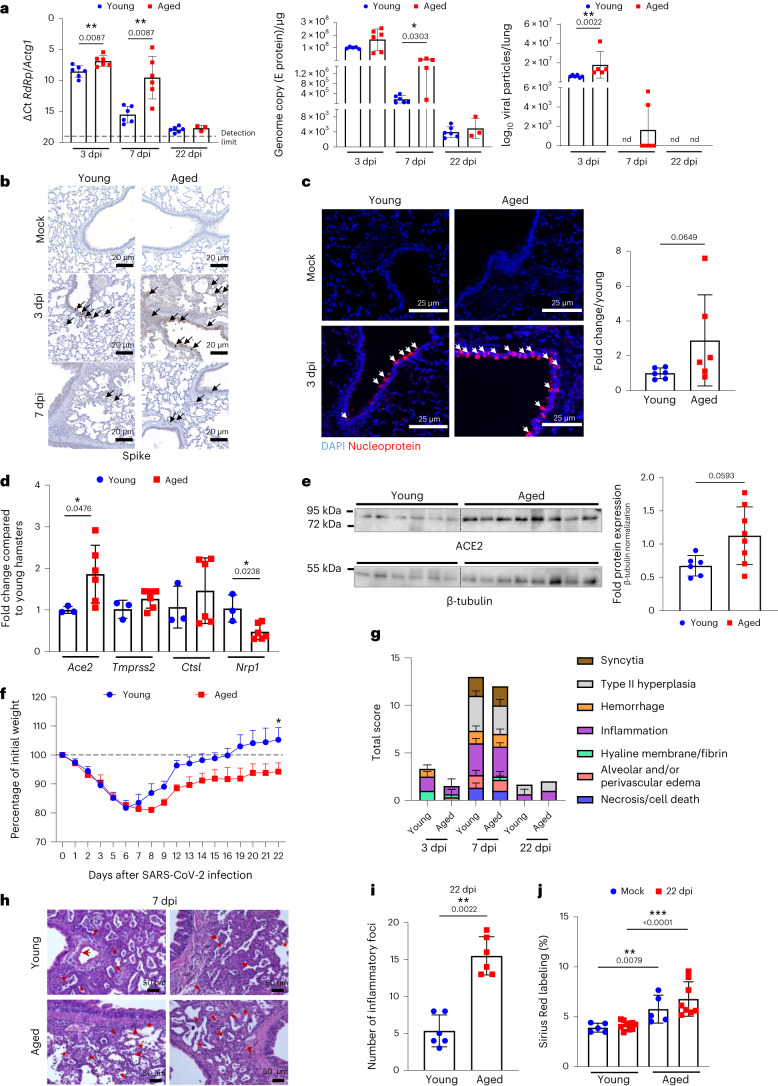
Effects of SARS-CoV-2 infection on aged hamsters. Young hamsters and aged hamsters were infected with SARS-CoV-2. Lungs were collected at 3 dpi, 7 dpi and 22 dpi (*n* = 3–6). Left and middle: quantification of viral RdRp and E protein transcript levels using RT–PCR assays. The data are expressed as Δ*C*t and genome copy per microgram of RNA. Right: number of infectious virus particles per lung (50% tissue culture infectious dose, TCID_50_). **b**, Immunohistochemistry analysis of spike. Scale bars, 20 μm. **c**, Leftl: immunofluorescence staining for DAPI (blue) and viral nucleoprotein (red) is shown. Scale bars, 25 μm. Right: the intensity of nucleoprotein signals was normalized by DAPI count. The histograms indicate the fold change relative to average intensity in young animals (*n* = 6). **d**, Gene expression was quantified by RT–PCR (fold change relative to average gene expression in young animals) (*n* = 3–6). **e**, Expression of AC2 and β-tubulin in young and aged whole lung homogenates as assessed by western blotting. Right: the relative protein levels normalized to β-tubulin are shown (*n* = 6–8). **f**, Body weight loss curves (four aged hamsters and six young hamsters). **g**, Histopathological examination of lung sections (H&E staining). The mean sum of the subscores is shown (*n* = 3–6). **h**, Representative photomicrographs at 7 dpi. Arrowhead: inflammatory cell infiltrate; star: alveolar wall rupture; sun: type II pneumocyte hyperplasia; thunderbolt: necrosis. Scale bars, 50 μm. **i**, Numbers of inflammatory foci per lung section (*n* = 6). **j**, Percentage of Sirius Red labeling (*n* = 5–10). **a**–**i**, One of two representative experiments is shown. **j**, A pool of two independent experiments is depicted. For all graphs, errors indicate mean ± s.d. Significant differences were determined using the two-tailed Mann–Whitney *U-*test (**a**,**c**,**d**,**e**,**g**,**i**) and the one-way ANOVA Kruskal–Wallis test (non-parametric), followed by Dunn’s post test (**j**). Significance of body weight regain (area under the curve) in infected young hamsters was calculated using the Wilcoxon matched-pairs signed-rank test (**f**). *P < 0.05, **P < 0.01, ***P < 0.001. Source data

**Extended Data Fig. 2:**
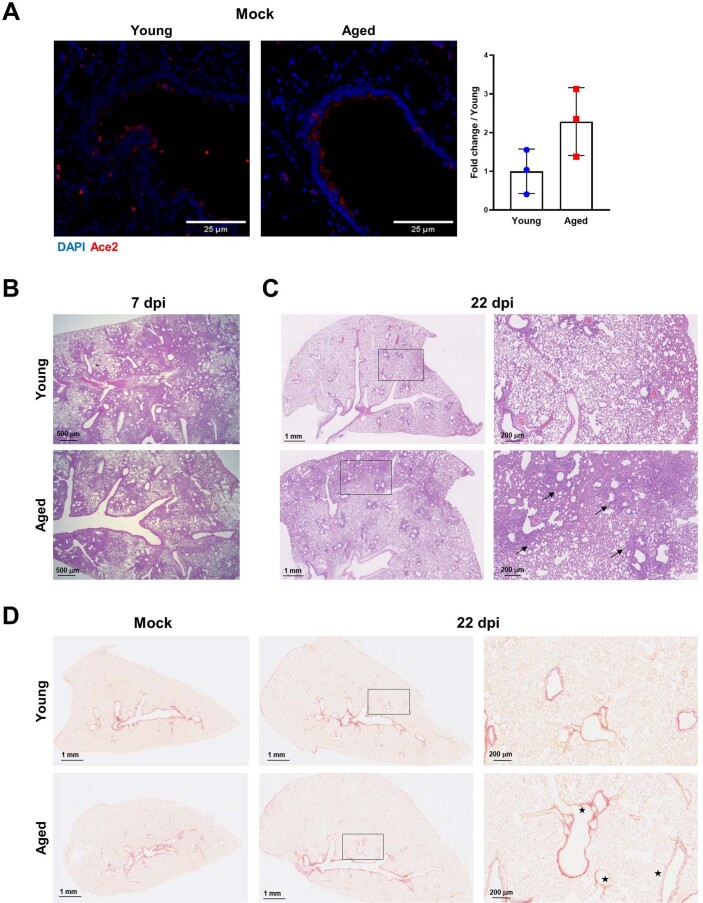
Ace2 expression in aged lungs and consequences of a SARS-CoV-2 infection on lung pathology. **A**, Immunofluorescence analysis of Ace2 in the lung from uninfected (mock) young hamsters and aged hamsters. Bars: 25 μm. Right panel, The histograms indicate the mean ± s.d fold change relative to average intensity in young animals (n = 3). Significant differences were determined using the two tailed Mann Whitney *U* test**. B** and **C**, Representative photomicrographs of lungs from SARS-CoV-2-^−^infected young hamsters and aged hamsters (H&E staining) at 7 dpi (**B**), and 22 dpi (**C**). **C**, Arrow: inflammatory cell infiltrate; sun: type II pneumocyte hyperplasia. **D**, The lungs were stained with Sirius Red. Representative images are depicted (22 dpi). Stars indicate altered structure of epithelial and vascular basal membranes. **B**-**D**, Scales are indicated. One of two representative experiments is shown.

The consequences of a SARS-CoV-2 infection in aged hamsters have rarely been addressed^41,42^. Relative to young hamsters, aged animals had higher expression of genes encoding RNA-dependent RNA polymerase (RdRp) and envelope (E) protein and viral particles at 3 and 7 days post-infection (dpi) (Fig. 2a). Immunohistochemistry (anti-spike) and immunofluorescence (nucleoprotein) staining on lung sections confirmed the higher viral load in aged animals (Fig. 2b,c). No major difference in viral antigen location (mostly in bronchial and alveolar epithelia) was observed between young and aged hamsters. Hence, SARS-CoV-2 replicated to higher levels in lung tissues of aged animals than in young animals. The levels of transcripts encoding ACE2 have been shown to be higher in aging human lung tissue relative to young human lung tissue^43,44^. In line, the steady-state expression of the *Ace2* gene was higher in aged hamster lungs relative to young hamster lungs (Fig. 2d). Regarding other cellular compounds involved in SARS-CoV-2 infection, transmembrane serine protease 2 (*Tmprss2*) and cathepsin L (*Ctsl*) transcript expression was unchanged in aged animals, whereas that of neuropilin-1 (*Nrp1*) was reduced. Enhanced ACE2 expression in aged lungs (mostly in alveolar and bronchiolar epithelia) was confirmed at the protein level by immunofluorescence and western blotting (Fig. 2e and Extended Data Fig. 2a).

With regard to morbidity, weight loss peaked at 6 dpi in young hamsters and at 8 dpi in aged hamsters. At 7 dpi and onwards, young animals started to recover body weight. The initial body weight was attained at 16 dpi. By contrast, the body weight increased in aged hamsters but at a much slower rate than in younger animals. Aged animals failed to recover their initial body weight at 22 dpi. It is noteworthy that one of the four aged hamsters suffered from respiratory distress at 9 dpi and so was euthanized. At 3 dpi, both age groups had developed bronchointerstitial pneumonia, congestion and intra-alveolar and interstitial cell infiltration. At 7 dpi, bronchoepithelial hyperplasia, bronchointerstitial pneumonia and perivascular and intra-alveolar mixed cell inflammation were markedly enhanced (Extended Data Fig. 2b). There were no clear age-dependent differences in the total disease score (Fig. 2g). However, small differences (mostly in the alveolar area) between aged hamsters and young hamsters were observed at 7 dpi. In fact, a loss of structure in the alveolar area (for example, alveolar wall rupture) was observed in aged animals (Fig. 2h). By contrast, the alveolar wall structure in young hamsters was essentially unaffected and was associated with marked alveolar epithelial cell hyperplasia. With regard to the longer-term consequences of a SARS-CoV-2 infection, the lungs still presented some areas of inflammation and type II hyperplasia at 22 dpi (Extended Data Fig. 2c). Interestingly, the number of these inflammatory foci was significantly higher in aged hamsters than in young hamsters (Fig. 2i). The intensity of Sirius Red staining indicated a higher collagen deposition in aged animals (Fig. 2j and Extended Data Fig. 2d). Interestingly, the structure of the basal membrane around bronchi and blood vessels was disorganized and sometimes disrupted in aged hamsters (Extended Data Fig. 2d, right panels). This alteration was not found in young hamsters. Overall, aged hamsters exhibited a higher viral load associated with a higher ACE2 expression at basal level and developed more intense post-acute sequelae in the lungs than their young counterparts did.

### ABT-263 reduces viral load and SASP factors in aged hamsters

**Fig. 3:**
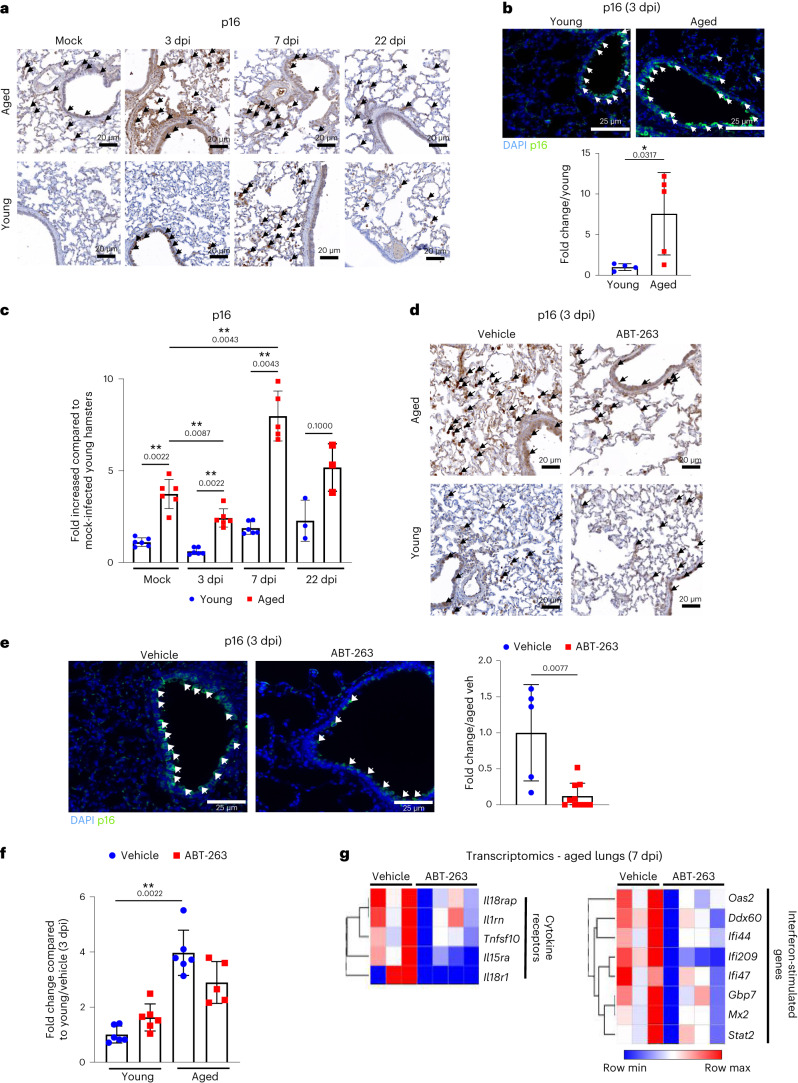
Effect of a SARS-CoV-2 infection on the frequency of pulmonary p16-positive cells and effects of ABT-263 treatment on p16 and SASP expression in lungs. **a**, Lungs from mock-infected and SARS-CoV-2-infected, aged hamsters and young hamsters were stained with a p16 antibody. Representative photomicrographs showing labeling of p16 are shown at 3 dpi, 7 dpi and 22 dpi. Scale bars, 20 μm. **b**, p16 labeling was performed on lung sections collected at 3 dpi. Scale bars, 25 μm. Bottom: the histograms indicate the fold change relative to average intensity in young animals (*n* = 4–5). **c**, The mRNA expression level of *Cdkn2a* (encoding p16) was quantified by RT–PCR. The data are expressed as fold increase relative to average gene expression in mock-infected young hamsters (*n* = 3–6). **d**, Aged hamsters and young hamsters were treated (or not) with ABT-263 and then infected with SARS-CoV-2. Arrows indicate p16-positive cells (3 dpi). Scale bars, 20 μm. **e**, Effect of ABT-263 treatment on p16 expression as assessed by immunofluorescence (3 dpi). Scale bars, 25 μm. Right: the intensity of p16 signals was normalized by DAPI count. The histograms indicate the fold change relative to average intensity in vehicle-treated infected, aged animals (*n* = 5–10). **f**, The *Cdkn2a* transcript levels are indicated. The data are expressed as the fold increase relative to average gene expression in vehicle-treated infected young hamsters (*n* = 5–6). **g**, Effect of ABT-263 treatment on the expression of genes related to SASP factors in infected, aged hamsters (*n* = 3–4, 7 dpi). Heat map (hierarchical clustering) of the differences in expression of SASP factors, calculated using the difference between log intensity of ABT-263 and the control (fold change > 1.5, *P* < 0.01). **a**–**f**, One of two representative experiments is shown. **g**, One experiment performed. For all graphs, errors indicate mean ± s.d. Significant differences were determined using the two-tailed Mann–Whitney *U-*test (**b**,**e**), moderated *t*-test after Benjamani–Hochberg correction (**g**) and one-way ANOVA Kruskal–Wallis test (non-parametric), followed by Dunn’s post test (**c**,**f**). *P < 0.05, **P < 0.01.

**Extended Data Fig. 3:**
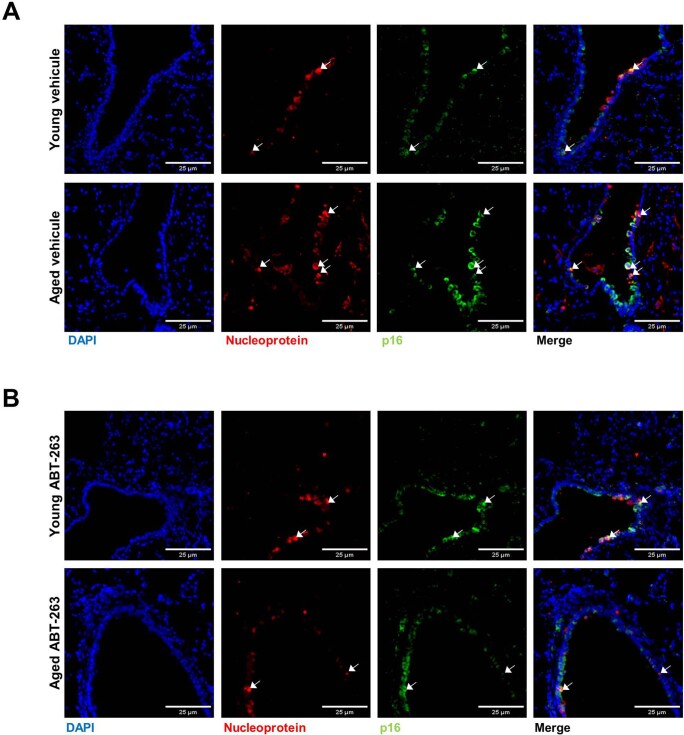
Virus antigen and p16 expression in vehicle-treated and ABT-263-treated young and aged lungs at 3 dpi. Immunofluorescence staining for DAPI (blue), viral nucleoprotein (red) and p16 (green) is shown. Labelling was performed on lung sections collected from vehicle-treated (**A**) and from ABT-263-treated (**B**) infected animals. Scales are indicated. One of two representative experiments is shown.

Respiratory viruses, including SARS-CoV-2, can induce cellular senescence in humans and in young animals^43,45–50^. We sought to determine whether SARS-CoV-2-induced lung cell senescence is exacerbated in aged animals. The number of p16-positive pulmonary cells increased during infection in aged hamsters, which was indicative of exacerbated lung senescence (Fig. 3a). Their numbers were higher in aged hamsters than in young hamsters (Fig. 3a,b). It is noteworthy that p16-positive cells were still visible at 22 dpi, albeit in lower numbers than at earlier timepoints (Fig. 3a). Some, but not all, p16-positive cells expressed virus antigen in young and aged hamsters (Extended Data Fig. 3a). In infected, aged animals, it is likely that pre-existing senescent cells (infected or not), virus-induced senescent cells (p16-positive and virus-positive cells) and secondary senescent cells (p16-positive and virus-negative cells)^31^ populate the lung tissue. Although transcript levels of *Cdkn2a* (which encodes p16) were not markedly enhanced during infection, its expression was higher in infected, aged hamsters relative to infected, young hamsters (Fig. 3c).

**Extended Data Fig. 4:**
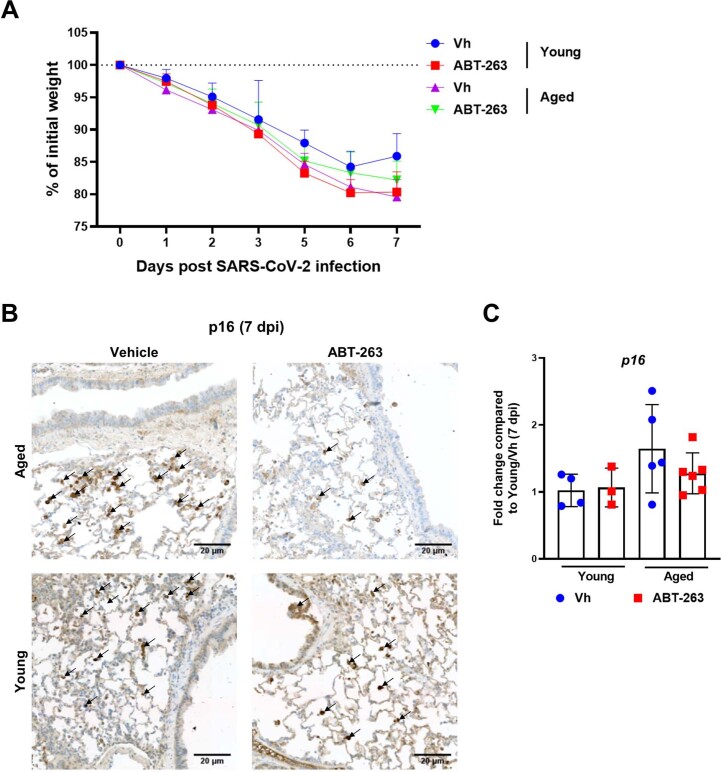
Effect of ABT-263 treatment on body weight loss and p16 expression at 7 dpi. Aged hamsters and young hamsters were treated (or not) with ABT-263 and then infected with SARS-CoV-2. **A**, Body weight loss of vehicle-treated and ABT-263-treated aged hamsters and young hamsters following a SARS-CoV-2 infection (n = 6). **B**, Effect of ABT-263 treatment on the frequency of p16-positive cells (7 dpi) as assessed by immunohistochemistry. Arrows indicate p16-positive cells. Bars: 20 μm. **C**, The p16 transcript levels are indicated (n = 3-6). The data are expressed as the fold increase relative to average gene expression in infected (7 dpi) vehicle-treated young hamsters (n = 3-6). For all graphs, errors indicate mean ± s.d. One of two representative experiments are shown (A-C). Significance of body weight loss between animal groups was calculated using wilcoxon matched-pairs signed rank test (**A**). Significant differences were determined using One-way ANOVA Kruskal-Wallis test (nonparametric), followed by the Dunn’s posttest test (**C**).

We next determined the effect of ABT-263 treatment in the context of infection. Hamsters were treated with ABT-263 1 day before SARS-CoV-2 infection and then daily until euthanization. No evidence of beneficial or adverse effects (for example, additional body weight loss) was observed during infection (Extended Data Fig. 4a). ABT-263 treatment depleted, albeit not completely, p16-positive cells in lung tissue from aged animals at both 3 dpi and 7 dpi (Fig. 3d,e and Extended Data Fig. 4b). In contrast, ABT-263 treatment failed to reduce the frequency of p16-positive cells in young animals. ABT-263 also reduced *Cdkn2a* transcript expression in infected, aged hamsters (Fig. 3f and Extended Data Fig. 4c). We then turned to analyze whether ABT-263 treatment results in reduced expression of SASP factors in infected, aged lungs. Transcriptomic analysis revealed that 134 genes, including genes related to SASP factors, were downmodulated after treatment with ABT-263 (fold change > 1.5, *P* < 0.01) (Fig. 3g). This included immune and inflammatory modulators, such as cytokines/cytokine receptors and interferon-stimulated genes (ISGs).

**Fig. 4:**
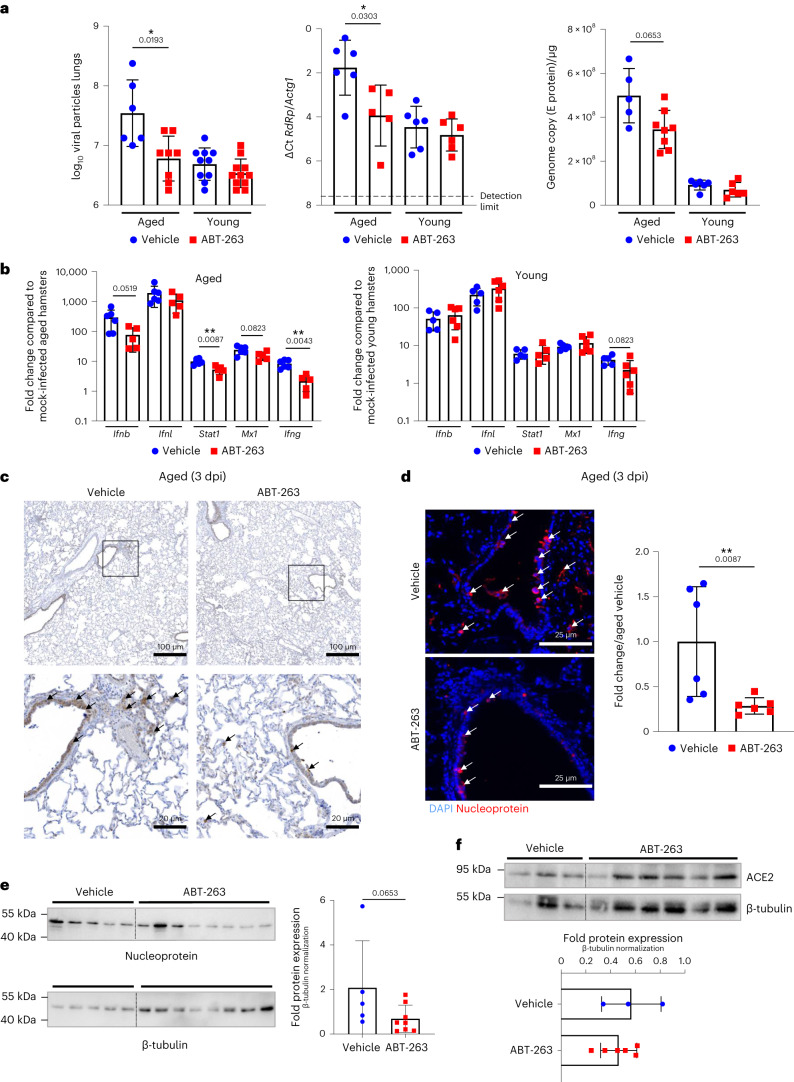
Effect of ABT-263 treatment on viral loads and ACE2 expression in lungs. Aged hamsters and young hamsters were treated (or not) with ABT-263 and then infected with SARS-CoV-2. Animals were euthanized at 3 dpi. **a**, Determination of viral loads in the lungs. Left: the number of infectious particles was determined in a TCID_50_ assay. The data are expressed as the number of infectious virus particles per lung (*n* = 6–11). Middle and right: quantification of viral RdRp and E protein transcript levels in the whole lungs, using RT–PCR. The data are expressed as Δ*C*t and genome copy per microgram of RNA (*n* = 5–8). **b**, mRNA copy numbers (for IFNs and ISGs) were quantified by RT–PCR. The data are expressed as the fold change relative to average gene expression in mock-infected animals (*n* = 5–6). **c**, Immunohistochemistry analysis of spike in the lung from SARS-CoV-2-infected, aged hamsters treated (or not) with ABT-263. Scale bars, 100 μm and 20 μm. **d**, Viral nucleoprotein labeling (immunofluorescence) was performed on lung sections. Scale bars, 25 μm. Right: the histograms indicate the fold change relative to average intensity in vehicle-treated infected, aged animals (*n* = 6). **e**,**f**, Expression of the viral nucleoprotein, ACE2 and β-tubulin (western blotting) in vehicle-treated and ABT-263-treated SARS-CoV-2-infected, aged hamsters (whole lung homogenates). The relative protein levels normalized to β-tubulin are shown (*n* = 3–8). For all graphs, errors indicate mean ± s.d. Pooled results from two independent experiments (**a**) and one of two representative experiments (**b**–**e**) are shown. Significant differences were determined using the two-tailed Mann–Whitney *U-*test (**b**,**d**,**e**,**f**) or one-way ANOVA Kruskal–Wallis test (non-parametric), followed by Dunn’s post test (**a**). *P < 0.05, **P < 0.01. Source data

**Extended Data Fig. 5:**
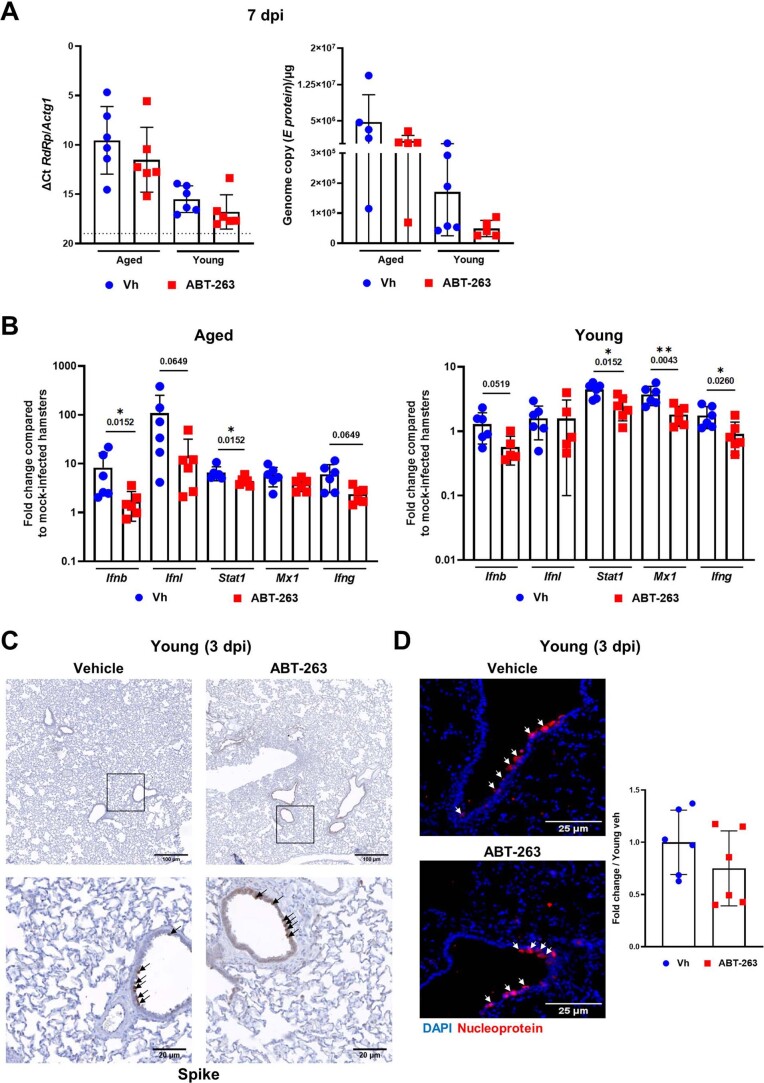
Effect of ABT-263 treatment on viral loads and ISGs. Aged hamsters and young hamsters were treated (or not) with ABT-263 and infected with SARS-CoV-2. **A**, Quantification of viral RdRp and E protein transcript levels in the whole lungs using RT-PCR assays (7 dpi). The data are expressed as ΔCt and genome copy/μg RNA (n = 5-6)**. B**, mRNA copy numbers of genes (interferons and ISGs), as quantified with RT-PCR. The data are expressed as the fold change, relative to average gene expression in mock-infected animals (n = 5-6). **C**, Immunohistochemistry analysis of spike in the lung from SARS-CoV-2-infected young hamsters treated (or not) with ABT-263 (3 dpi). Bars: 100 μm and 20 μm. **D**, Nucleoprotein labelling (immunofluorescence) was performed on lung sections collected at 3 dpi. Bars: 25 μm. Right panel, The histograms indicate the fold change relative to average intensity in vehicle-treated young animals (n = 6). **A-D**, One of two representative experiments is shown. For all graphs, errors indicate mean ± s.d. Significant differences were determined using the two tailed Mann Whitney *U* test (**B** and **D**) and One-way ANOVA Kruskal-Wallis test (nonparametric), followed by the Dunn’s posttest test (**A**). * *P* < 0.05, ** *P* < 0.01.

**Extended Data Fig. 6:**
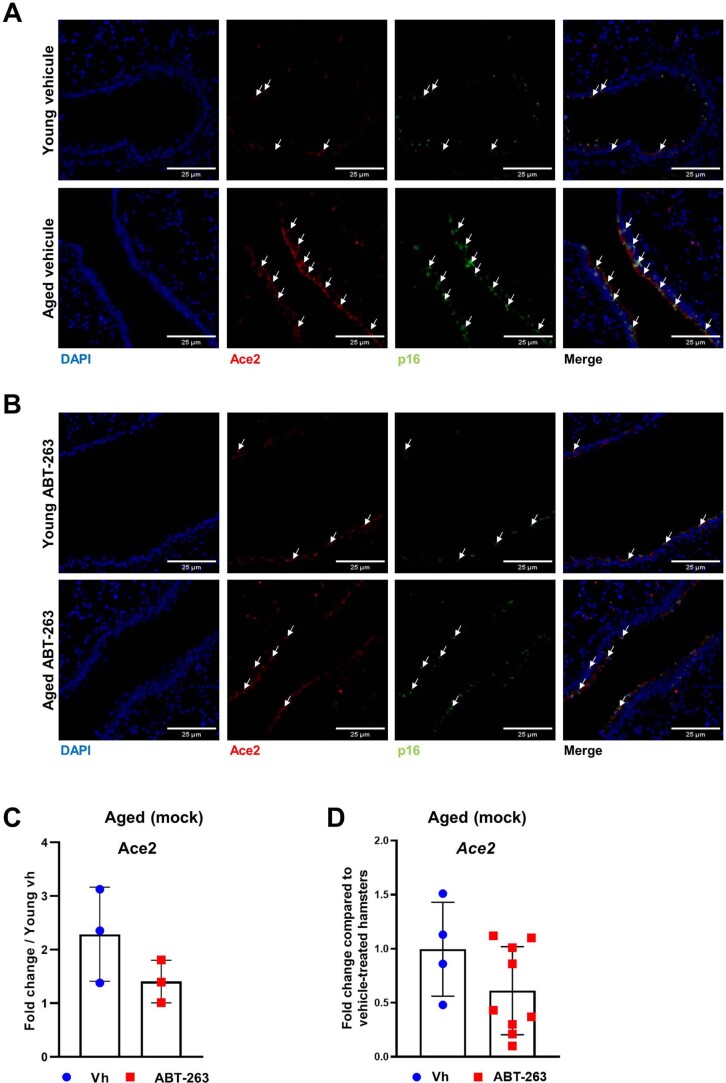
Ace2 and p16 expression in vehicle-treated and ABT-263-treated young and aged lungs. Lungs were collected from uninfected animals previously treated or not with ABT-263. Immunofluorescence staining for DAPI (blue), Ace2 (red) and p16 (green) is shown. Labelling was performed on lung sections collected from vehicle-treated (**A**) and from ABT-263-treated (**B**) animals. Scales are indicated. **C**, The intensity of Ace2 signals was normalized by DAPI count. The histograms indicate the fold change relative to average intensity in vehicle-treated young hamsters (n = 3). **D**, mRNA copy numbers of Ace2 were quantified in RT-PCR assays (n = 4-9). The data are expressed as the fold change relative to average gene expression in vehicle-treated aged hamsters. **A-D**, One of two representative experiments is shown. For all graphs, errors indicate mean ± s.d. Significant differences were determined using the two tailed Mann Whitney *U* test.

We next sought to determine whether clearing senescent cells with ABT-263 would impact viral replication during SARS-CoV-2 infection. ABT-263 lowered pulmonary viral particles in aged hamsters, but not young hamsters, at 3 dpi (Fig. 4a). These data were confirmed by quantitative RT–PCR assays (transcripts for RdRp and E protein). No clear differences were observed in viral mRNA load at 7 dpi (Extended Data Fig. 5a). The mRNA expression level of genes encoding interferons (IFNs) and ISGs generally mirrored the viral load at 3 dpi and onwards. ABT-263 treatment of aged hamsters (but not of young hamsters) was associated with lower mRNA expression of IFNs and ISGs at 3 dpi (Fig. 4b) and, to a lesser extent, at 7 dpi (Extended Data Fig. 5b). In line with the above data, the number of spike and nucleoprotein-positive cells was lower in aged (but in not young) hamsters treated with ABT-263 (Fig. 4c,d and Extended Data Fig. 5c,d for young animals). Extended Data Fig. 3b shows that p16 and virus antigen double-positive cells were preferentially eliminated by ABT-263, whereas most virus-positive and p16-negative cells were not removed by the drug. Lastly, western blots of viral nucleoprotein also indicated that the viral load was significantly lower in ABT-263-treated aged hamsters than in the control group (Fig. 4e). To investigate whether the reduced viral load associates with reduced ACE2 expression, quantitative RT–PCR, western blotting (whole lungs) and immunofluorescence on lung sections were performed on uninfected animals. Although it did not reach significance, ACE2 transcript and protein expression was reduced upon ABT-263 treatment (Fig. 4f and Extended Data Fig. 6). The number of double-positive (ACE2 and p16) cells was reduced upon ABT-263 treatment. We conclude that the pre-infection depletion of (ACE2-expressing) senescent cells in aged hamsters reduces the viral load in the lungs.

### ABT-263 ameliorates acute and long-term pulmonary disease

**Fig. 5:**
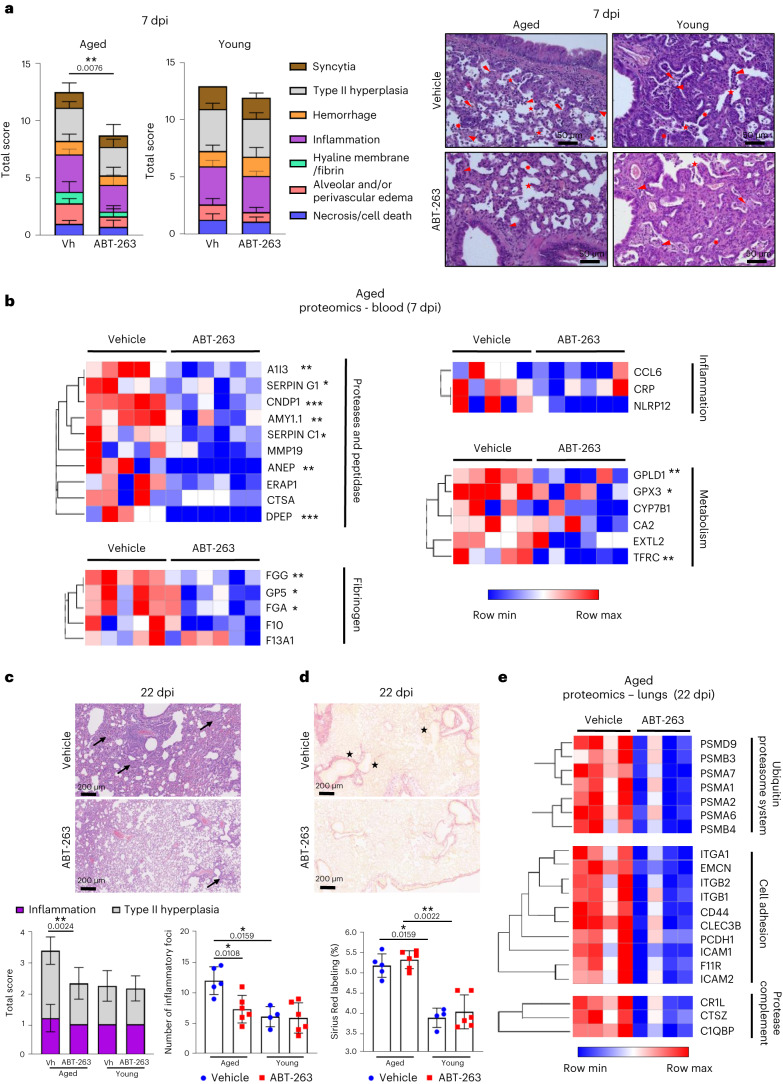
Effect of ABT-263 treatment on pulmonary and systemic inflammation in aged hamsters. Aged hamsters and young hamsters were treated (or not) with ABT-263 and then infected with SARS-CoV-2. Animals were euthanized at 7 dpi and 22 dpi. **a**, Left: histopathological examination of lung sections (H&E staining, 7 dpi). The sum of the subscores is shown (*n* = 11–12 aged and *n* = 6 young). Right: photomicrographs showing lower alveolar destruction in ABT-263-treated aged hamsters (but not ABT-263-treated young hamsters). Arrowhead: inflammatory cell infiltrate; star: alveolar wall rupture; sun: type II pneumocyte hyperplasia; thunderbolt: necrosis; arrow: activated blood vessel. Scale bars, 50 μm. **b**, Heat maps of the differential expressed prothrombotic and inflammatory factors in the serum of vehicle-treated and ABT-263-treated aged hamsters, in a mass spectrometry analysis of the proteome (fold change in protein level > 1.2, *P* < 0.05) (*n* = 5–6). **c**, Histopathological examination of lung sections (H&E staining, 22 dpi). Lower panels: The total histology score (left) and the numbers of inflammatory foci (inflammation and type II hyperplasia) per lung section (right) are shown (*n* = 4–6). **d**, Sirius Red labeling in the lungs of vehicle-treated and ABT-263-treated aged hamsters and young hamsters at 22 dpi. Top: representative images showing (stars) the destructured basal membranes in vehicle-treated aged animals. Bottom: the percentages of Sirius Red labeling are shown (*n* = 4–6). **e**, TMT-based proteomic analysis of lung extracts (vehicle-treated and ABT-263-treated aged hamsters). Heat maps of the differentially expressed components, in a mass spectrometry analysis of the proteome, are depicted (fold change in protein abundance > 2, *P* < 0.05) (*n* = 4). For all graphs, errors indicate mean ± s.d. Pooled results from two independent experiments (**a**, left) and one of two representative experiments (**a**, right, and **b**–**e**) are shown. Significant differences were determined using the two-tailed Mann–Whitney *U-*test (**a**,**b**,**e**) and one-way ANOVA Kruskal–Wallis test (non-parametric), followed by Dunn’s post test (**c**,**d**). *P < 0.05, **P < 0.01, ***P < 0.001.

**Extended Data Fig. 7:**
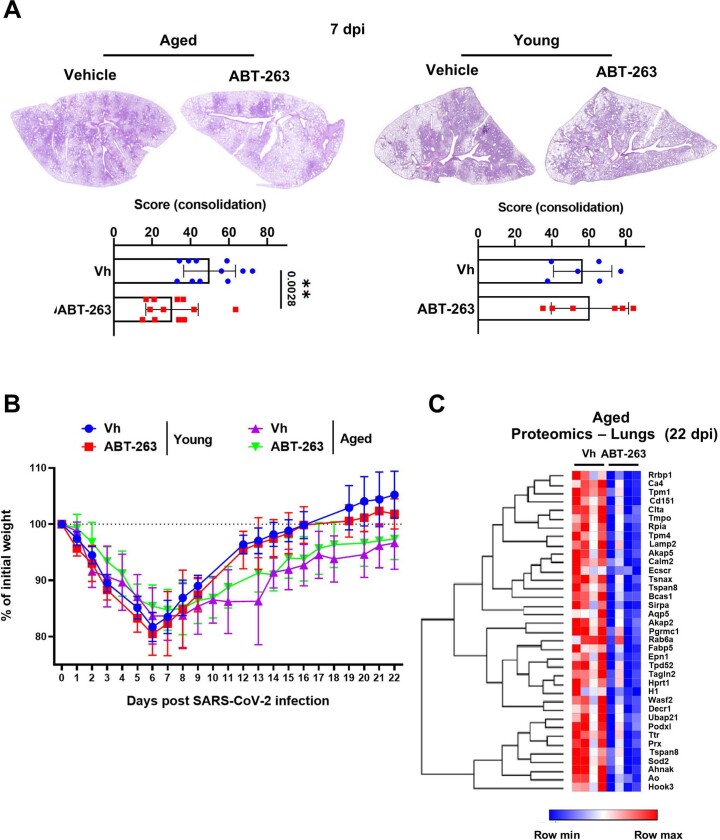
Effect of ABT-263 treatment on lung pathology and body weight loss during a SARS-CoV-2 infection. Aged hamsters and young hamsters were treated (or not) with ABT-263 and then infected with SARS-CoV-2. **A**, Representative micrographs showing H&E-stained lungs from aged hamsters and young hamsters treated (or not) with ABT-263 (magnification: x 2.5). The percentage of lung sections affected by subacute bronchointerstitial pneumonia (consolidation) is depicted for each group of animals (lower panel, (n = 11-12 aged and n = 6 young). **B**, Body weight loss curves for infected animals (n = 5-6). **C**, Heatmaps of the differential expressed components in the lungs of vehicle-treated and ABT-263-treated aged hamsters, in a mass-spectrometry analysis of the proteome (fold change in protein abundance >2, *P* < 0.05) (n = 4). Pooled results from two independent experiments (A left, B and C) and one of two representative experiments (A right) are shown. For all graphs, errors indicate mean ± SD. **A** and **C**, Significant differences were determined using the two tailed Mann Whitney *U* test. ** *P* < 0.01.

We next assessed the impact of ABT-263 treatment on COVID-19-like lung disease. At 7 dpi, the surface of lung sections affected by subacute bronchointerstitial pneumonia was significantly lower in ABT-263-treated aged animals than in the control group (Extended Data Fig. 7a). In young animals, ABT-263 had no effect on pneumonia. The total histological score indicated that the disease was less intense in ABT-263-treated aged animals than in the control group (Fig. 5a, left panels). More specifically, inflammation (inflammatory infiltrates), hemorrhage, syncytia and alveolar destruction (less alveolar wall rupture; Fig. 5a, right panels) were markedly less intense or widespread in ABT-263-treated aged animals. In contrast, the total histology score in ABT-263-treated young hamsters did not reveal any overt differences regarding COVID-19-like parameters, including alveolar destruction (Fig. 5a). We next wondered whether ABT-263 treatment influenced circulating levels of biomarkers of SARS-CoV-2 infection. Given that these changes are difficult to evidence with ELISAs, we used mass spectrometry to analyze the proteome. At 7 dpi, the serum concentrations of a number of SASP-related factors were lower in ABT-263-treated aged hamsters than in the control group (fold change > 1.2, *P* < 0.2) (Fig. 5b). Some of the observed differences in levels of prothrombotic and inflammatory factors are reportedly correlated with the severity of COVID-19 in humans^51,52^. The prothrombotic factors included proteases, protease inhibitors, peptidases and fibrinogen family members, and the inflammatory factors included C-reactive protein and the monocyte–macrophage chemoattractant chemokine (C-C motif) ligand 6. Treatment with ABT-263 was also associated with lower levels of enzymes and transporters from various metabolic pathways.

We then investigated whether ABT-263 treatment effects on the longer-term consequences of a SARS-CoV-2 infection (22 dpi). ABT-263 did not significantly accelerate the body weight recovery in infected, aged hamsters (Extended Data Fig. 7b). Of note, one aged hamster treated with vehicle out of six died at 14 dpi, whereas all ABT-263-treated animals survived infection. Interestingly, relative to the vehicle control, histological examination (hematoxylin and eosin (H&E) staining) at 22 dpi revealed lower areas of inflammation and type II hyperplasia in ABT-263-treated aged hamsters relative to controls (Fig. 5c). The number of inflammatory foci was significantly reduced in aged animals after ABT-263 treatment. No effect was evidenced in young hamsters. The percentage of Sirius Red staining was equivalent in vehicle-treated and ABT-263-treated aged hamsters, indicating no change on collagen deposition (Fig. 5d). However, the basal membrane was less disorganized and disrupted (indicated by stars in Fig. 5d) in ABT-263-treated aged hamsters relative to controls. To examine whether ABT-263 treatment in aged animals impacted on the expression of components involved in chronic lung diseases, we performed a proteomic analysis on lung homogenates. We observed that most altered proteins with a fold change greather than 2 had a reduced expression in ABT-263-treated animals relative to vehicle controls (58 downregulated and six upregulated, *P* < 0.05) (Fig. 5e and Extended Data Fig. 5c). Among downregulated proteins, a large proportion belonged to the ubiquitin–proteasome system, the uncontroled activity of which plays a part in chronic lung diseases^53,54^ (Fig. 5e). The expression of cell adhesion molecules is dysregulated during chronic lung diseases^55,56^. The expression of members of the immunoglobulin superfamily (ICAM-1, ICAM-2 and F11R) and integrins (ITGA1, ITGB1 and ITGB2) was reduced in ABT-263-treated animals compared to the control group (Fig. 5e). Although some proteases (for example, cathepsin) and members of the complement pathway (C1q-binding protein and complement component receptor 1) were also downregulated in ABT-263-treated animals, classical markers of fibrosis, such as extracellular matrix proteins and growth factors, were not significantly altered (Fig. 5e and Extended Data Fig. 7c). Taken as a whole, depletion of senescent cells ameliorates acute lung and systemic COVID-19-like disease and mitigates longer-term consequences of a SARS-CoV-2 infection in aged hamsters.

## Discussion

Only a few studies have addressed the effect of advanced age on experimental SARS-CoV-2 infections in hamsters^41,42,57–60^. With the exception of Selvaraj et al.^41^ and Bogard et al.^42^, these studies used middle-aged animals, aged from 5 to 8 months. As the life expectancy of golden hamsters is typically 20–24 months, we chose to study 22-month-old hamsters (equivalent to ~80-year-old humans). Unlike the findings of Osterrieder et al.^58^ but in line with the report by Griffin et al.^59^ (in middle-aged animals), we found that the viral load in lungs was higher in aged hamsters than in young hamsters. Of note, Selvaraj et al.^41^ also observed a prolonged period of active virus replication in the upper respiratory tract of middle-aged and aged hamsters. It is noteworthy that the load of mouse-adapted SARS-CoV-2 was reportedly greater in middle-aged (12-month-old) mice than in young mice^61–63^. The higher viral load in old/aged animals might be due to greater viral entry, faster replication and/or a weaker or slower antiviral response (for example, type I and type III IFNs)^43,63^. In line with other studies^57–59^, the age-dependent differences in the severity of lung lesions observed during the early (3 dpi) and acute (7 dpi) responses were not large. Our present data showed that aged hamsters had developed moderate fibrosis (collagen deposition) in the lungs at 22 dpi and, in contrast to young animals, failed to recover their initial body weight, which is indicative of long-term sequelae.

Compared to young animals, our data suggest greater cellular senescence in the lungs of uninfected, aged animals as assessed by p16 and BCL-XL protein expression and SA-β-Gal activity. The expression of p16 was enhanced during SARS-CoV-2 infection, in line with other reports^45,46,48^, and remained more elevated in aged animals relative to young counterparts. The causes for enhanced p16 expression, and in general of SARS-CoV-2-induced senescence, are still unknown. It probably relies on various molecular pathways originating from virus propagation (for example, reactive oxygen species production by stressed mitochondria) or specific viral molecular components^12^. Indirect effects may also occur because the SASP is known to induce paracrine senescence of neighboring cells during a SARS-CoV-2 infection^45,48,64^. Next, we assessed the effect of treatment with ABT-263. We chose to treat animals just before the viral infection (so that pre-existing senescent cells were removed) and then continue the treatment until euthanization to eliminate virus-induced senescent cells. In our setting, ABT-263 treatment partially eliminated p16-positive cells and decreased the senescence-associated signature in SARS-CoV-2-infected, aged animals. We next assessed viral loads in hamster lung tissues in the context of ABT-263 treatment. We did not make any starting hypotheses in this respect because senescent cells could have potentially opposing effects on the viral load. Indeed, SASP factors might suppress viral replication (via IFN-related and/or chemokine pathways) and/or promote viral replication (via immunosuppressive factors and/or enhanced expression of viral receptors)^31,50,65–70^. Because pre-existing senescent cells might be preferential targets for SARS-CoV-2 (due to high levels of ACE2 expression on epithelial cells and/or elevated permissiveness for virus replication)^71,72^, one would expect the removal of senescent cells by ABT-263 to reduce the viral load in aged hamsters. Indeed, our data evidenced a lower viral load in lungs from ABT263-treated aged animals but not in lungs from ABT263-treated young animals; the latter finding (young animals) is in line with the reports by Lee et al.^45^ and Tsuji et al.^48^. Our results are also in line with the report of Camell et al.^31^ of a lower virus (mouse hepatitis virus) load in senolytic-treated, aged mice (>20 months of age). Hence, targeting senescent cells in aged hamsters results in a lower viral load. The lack of an antiviral effect of ABT-263 in young hamsters might be due (1) to inefficient removal of virus-induced senescent cells in this system (in line with ref. ^48^), and/or (2) to the fact that cellular senescence in young hamster lungs starts when the viral load is already high (for virus kinetics^33^). We hypothesize that, in aged animals, targeting pre-existing senescent cells, rather than stress (virus)-induced senescent cells, with ABT-263 results in a lower viral load in vivo. The mechanisms behind this effect have yet to be determined and warrant investigation in the future. A plausible explanation is that ABT-263 acts at the early stage—namely, attachment and entry—of the virus replication by eliminating AC2-positive cells. Our data indeed showed a reduced AC2 expression and a lower number of ACE2 and p16 co-expressing cells after ABT-263 treatment in aged hamsters. ABT-263 may also impact on other steps of viral replication in senescent cells, such as transcription, replication, gene expression, assembly, maturation and release, for instance by modulating the endoplasmic reticulum stress pathway^73^. Other alternative and/or additional mechanisms are also plausible. For instance, dying senescent cells targeted by ABT-263 may release danger, antiviral signals, such as IFN inducers. Further studies will be necessary to address these key questions. Notably, ABT-263 treatment also reduced the severity of lung disease (acute phase) in SARS-CoV-2-infected, aged hamsters. Again, it was difficult to form starting hypotheses. On the one hand, epithelial cells are prone to cellular senescence in old lungs^74^, and so eliminating these cells might have led to substantial effects, including pulmonary barrier leakage. Moreover, an ABT-263-induced increase in the apoptosis of senescent cells in the lungs might have accentuated the local inflammatory response markedly. On the other hand, one might expect ABT-263 treatment to have beneficial effects. Camell et al.^31^ reported that SASP factors derived from senescent cells (in vitro chemical induction) could amplify inflammation and the trafficking of macrophages (considered to be harmful in COVID-19)^31^. SARS-CoV-2 infection of pre-existing senescent cells may amplify their pro-inflammatory SASP. Accordingly, treatment with ABT-263 ameliorated systemic health parameters by substantially lowering serum concentrations of SASP-related compounds in general and those involved in thrombosis and inflammation in particular. Our data also showed that early treatment with ABT-263 ameliorates sequelae of a SARS-CoV-2 infection as measured by reduced type II hyperplasia, more organized basal membrane and lowered expression of factors known to play a part in chronic lung diseases^53–56^. This included members of the ubiquitin–proteasome system (a major protein degradation system) and cell adhesion molecules. On the other, ABT-263 treatment did not ameliorate body weight recovery and collagen deposition in lungs. In agreement with the reports from Tsuji et al.^48^ and (to a lesser extent) Lee et al.^45^ (who observed slight differences), we did not observe a positive effect of ABT-263 treatment on lung disease in young hamsters. As suggested by Tsuji et al.^48^, this finding could be explained by the lack of efficiency of ABT-263 in infected young hamsters—a situation not observed in mice^45,48^. These apparently contradictory findings might be due to differences in the pathways regulating the survival of age-associated pre-existing and virus-induced senescent cells in hamsters versus in mice.

The present study had some limitations. Because there are no p16 knockout hamsters, we cannot rule out the possibility that the results of tissue staining with the p16 antibody used in the current study detect a non-specific signal rather than p16. Although our results provide insights into therapies that target senescent cells and might improve COVID-19 outcomes in older individuals, golden hamster data should be interpreted with caution. Indeed, this model does not replicate all the features of severe COVID-19; the hamster is infected by SARS-CoV-2 but survives and rapidly resolves the COVID-19-like disease, although post-acute sequelae persist^75^. Moreover, the extrapulmonary disorders and organ damage seen in hamsters are not the same as those found in humans during severe COVID-19, although some similarities (blood levels of SASP factors) were noted in the present work. In the present study, we focused on an early senolytic intervention and, thus, depletion of the resident senescent cells before infection (for example, to reduce cell targets for SARS-CoV-2 and alleviate the SASP). The effects of post-infection ABT-263 treatment in aged animals remain to be investigated. Another limitation of the present study relates to our focus on ABT-263, a drug that targets BCL-2 family proteins. The specific targeting of senescent cells should now be studied using other classes of senolytics. The senotherapeutic potential of dasatinib (a tyrosine kinase inhibitor) and quercetin (a kinase-inhibiting flavonoid), which were recently shown to alleviate cellular senescence-associated (lung) diseases in human^76,77^, should be investigated in our settings. It is noteworthy that the combination of dasatinib and quercetin reduced lung pathology in preclinical models of COVID-19 (ref. ^45^). In conclusion, our present results describe the causative role of senescent cells in COVID-19-like disease in aged hamsters and, thus, highlight potential mechanisms by which advanced age influences COVID-19. Given that an accumulation of senescent cells is observed in many chronic diseases (such as obesity, diabetes and pulmonary disorders), our present results suggest that senolytics might protect individuals with a greater risk of poor COVID-19 outcomes. Several clinical trials to determine whether natural senolytics (for example, fisetin and quercetin, alone or in combination with other drugs) have an effect on COVID-19 in older adults and adults with comorbidities are in progress^78–81^. If these clinical trials are successful (some encouraging early results have been described), our study results would suggest that the depletion of senescent cells is associated with better clinical outcomes in older patients with COVID-19 and would validate the ‘geroscience’ hypothesis by positing senolytics as effective drugs against acute (respiratory) viral diseases in older adults^28,31^.

## Methods

### Animals, infections and ethics

Young (2-month-old) and aged (22-month-old) male Syrian golden hamsters (*Mesocricetus auratus*), equivalent to young adult (~20 years old) and aged (~80 years old) humans, respectively, were purchased from Janvier Laboratory. Animals were infected with 100 µl of DMEM containing (or not, for mock (control) animals) 2 × 10^4^ TCID_50_ (50% of the tissue culture infectious dose) of SARS-CoV-2 (hCoV-19_IPL_France strain of SARS-CoV-2)^33,42^. For tissue collection, animals were euthanized with an intraperitoneal injection of euthasol (140 mg kg^−1^). Lungs were collected from non-infected (mock) hamsters and from SARS-CoV-2-infected hamsters at 3 dpi, 7 dpi and 22 dpi. Two right lobes of the lung were used to quantify the viral load, and the other two right lobes were used for gene expression analyses. The left lobe was kept for histologic analyses. All experiments involving SARS-CoV-2 were performed within the Biosafety Level 3 facility of the Institut Pasteur de Lille. The protocols were validated by the local committee for the evaluation of the biological risks and complied with current national and institutional regulations and ethical guidelines (Institut Pasteur de Lille/B59-350009). The experimental protocols using animals were approved by the institutional ethical committee, Comité d’Ethique en Experimentation Animale (CEEA) 75, Nord Pas-de-Calais. The animal study was authorized by the Education, Research and and Innovation Ministry under registration number APAFIS#25041-2020040917227851v3.

### Treatment with ABT-263

ABT-263 (CliniSciences) was dissolved in DMSO for stock preparation and kept at −20 °C. For in vivo treatments, 10% ABT-263 was formulated in 30% polyethylene glycol 400 and 60% Phosal 50 PG. To assess the potential efficacy of ABT-263 treatment in non-infected animals, aged hamsters were administered by oral gavage (200 μl) with ABT-263 (65 mg kg^−1^) or vehicle (DMSO) control for three consecutive days. To assess the potential efficacy of ABT-263 treatment in SARS-CoV-2-infected animals, young hamsters and aged hamsters were administered with ABT-263 (65 mg kg^−1^) or vehicle (DMSO) 1 d before infection and then daily until 6 dpi.

### Determination of β-Gal activity

SA-β-Gal staining on lung sections was performed as described^39^. In brief, lungs were fixed in 2% formaldehyde and 0.2% glutaraldehyde in PBS for 30 min at room temperature. Tissues were embedded in optimal cutting temperature (OCT) compound and cut into 5-µm sections. The sections were stained as described^39^. Images were acquired using the EVOS M5000 Imaging System (Thermo Fisher Scientific). Alternatively, lung cell suspensions^39^ were used to quantify β-Gal activity. Cells (3 × 10^6^ per milliliter) were resuspended in phosphate buffer (pH 6.0) and subjected to repeated freeze–thaw cycles. Cells were centrifuged at 12,000*g* for 7 min, and supernatants were mixed with 2-nitrophenyl-β-d-galactopyranoside (ONPG) (2.2 mg ml^−1^) and 1 mM MgCl_2_ in phosphate buffer. After overnight incubation, two volumes of 1 M sodium carbonate were added, and absorbance was measured at 450 nm (Thermo Fisher Scientific, Multiskan FC).

### Expression of hamster p16 in HeLa cells

The coding sequence of hamster *Cdkn2a* was amplified by RT–PCR from RNA extracted from aged hamster lungs using primers shown in Table 2 (Eurofins Scientifics). The PCR product was inserted in plasmid pcDNA3.1(+) (Invitrogen). The construct was confirmed by sequencing. HeLa cells (American Type Culture Collection, CCL-2) grown on glass coverslips were transfected with pcDNA-p16 or pcDNA3.1 as a control using the TransIT-LT1 transfection agent (Mirus Bio). Transfected cells were cultured for 48 h, fixed with 4% paraformaldehyde and processed for immunofluorescence detection of p16 using a rabbit antibody from Abcam (ab211542, 1:100) or with a mouse monoclonal antibody from Santa Cruz Biotechnology (sc377412, 1:100) and an Alexa Fluor 488 (1:800, Invitrogen) as a secondary antibody.

**Table 2:** Sequences of oligonucleotides used in the present study

Oligonucleotides	
*Wdr63*	Forward 5′-GAGAGGGTCCACTTTTCGGG-3′
	Reverse 5′-GGCAGCCAGTGTATGTCTGT-3′
*Fank1*	Forward 5′-TCGATCGGCAGATACCACAG-3′
	Reverse 5′-CCCAAGAAGCTCCGTGTCTT-3′
*Fgf9*	Forward 5′-ACGAGAAGGGGGAGCTGTAT-3′
	Reverse 5′-CGGGTCCACTGGTCTAGGTA-3′
*Adam8*	Forward 5′-CATCAGACTCTTGCTTTGCC-3′
	Reverse 5′-AAACCTTCCCCTCCTCACAC-3′
*SerpinB10*	Forward 5′-AGAACTTGCATCCTCCAGAAA-3′
	Reverse 5′-CTGCGGTTCTGCACCAAAAT-3′
*Cdkn2a*	Forward 5′-ACAGTATCTACGTGCGGCTG-3′
	Reverse 5′-TGTCTGGGCAGAAGTTACGC-3′
*Cdkn1a*	Forward 5′-AGCGATATTGTTCGGAGGCA-3′
	Reverse 5′-CACTTTGCTCCTGTGTGGGA-3′
*Bcl2*	Forward 5′-CTTTGAGTTCGGTGGGGTCA-3′
	Reverse 5′-GGTCGGTGAACCTCACTTGT-3′
*Ace2*	Forward 5′-CTGGGAAAACTCCATGCTG-3′
	Reverse 5′-GAACGATCTCTCGCTTCATCT-3′
*Tmprss2*	Forward 5′-GGGCTACGAGCTTTATGAAGC-3′
	Reverse 5′-GGACGAACAGGAGTCACTGTG-3′
*Ctsl*	Forward 5′-GGGTGATGTCCCCAAGTCTG-3′
	Reverse 5′-TGGCCACAGCCTTCATTAGG-3′
*Nrp1*	Forward 5′-CTGGAAAGAAGGGCGTGTCT-3′
	Reverse 5′-CTTCATATCCGGGGGTGCTC-3′
*Ifnb*	Forward 5′-ACCCTAAAGGAAGTGCCAG-3′
	Reverse 5′-CCAGCTGCCAGTAATAGCTC-3′
*Ifnl*	Forward 5′-CCCACCAGATGCAAAGGATT-3′
	Reverse 5′-CTTGAGCAGCCACTCTTCTATG-3′
*Stat1*	Forward 5′-TCCATGCGGTTGAACCCTAC-3′
	Reverse 5′-TGTCAGTGTTCTGTGCTCACTT-3′
*Mx1*	Forward 5′-GGTATCGTTACCAGGTGCCC-3′
	Reverse 5′-GGTCTGGAACACTTGGGGAG-3′
*Ifng*	Forward 5′-TGTTGCTCTGCCTCACTCAGG-3′
	Reverse 5′-AAGACGAGGTCCCCTCCATTC-3′
*Actg1*	Forward 5′-ACAGAGAGAAGATGACGCAGATAATG-3′
	Reverse 5′-GCCTGAATGGCCACGTACA-3′
*RdRp*	Forward 5′-GTGARATGGTCATGTGTGGCGG-3′
	Reverse 5′-CARATGTTAAASACACTATTAGCATA-3′
Envelope (E) gene	Forward 5′-ACAGGTACGTTAATAGTTAATAGCGT-3′
	Reverse 5′-ATATTGCAGCAGTACGCACACA-3′
	Probe FAM-ACACTAGCCATC-CTTACTGCGCTTCG-MGB
*Cdkn2a* (protein expression)	Forward 5′-TTTGAAGCTTGCCACCATGGAGCCCTCTGCGGAC-3′
	Reverse 5′- TTTGGGATCCTTAGTAGGGCCCTAGGGGGTG -3′

### Determination of the viral load

The load of live, infectious viruses and the amount of viral RNA were measured using the Reed–Muench TCID_50_ assay and quantitative RT–PCR assays, respectively, as described^33^. Specific primers (Eurofins Scientifics) are shown in Table 2. Individual mRNAs were quantified relative to expression of the genes encoding RdRp and gamma actin (*Actg1*). The viral load was expressed as the amount of viral RNA relative to the *Actg1* expression level (Δ*C*t). Viral load quantification (genomic and mostly subgenomic) of hamster tissues was assessed as follows. One-step quantitative PCR assay was performed using Takyon Low Rox one-step RT probe Mastermix (Eurogentec) and specific primers and probe targeting the envelope (E) gene (Table 2). A synthetic gene containing the SARS-CoV-2 envelope gene was used to construct the standard curve^42^. Viral load quantification was assessed by linear regression using a standard curve of six known quantities of plasmids containing the envelope sequence (ranging from 10^7^ to 100 copies). The threshold of detection was established as 200 viral copies per microgram of RNA. Viral protein in lung tissue was quantified by western blotting, as follows.

### Western blotting

Lung or cell extracts were lysed in RIPA buffer (50 mM Tris-HCl pH 8, 150 mM NaCl, 1% NP-40, 0.5% sodium deoxycholate and 0.1% SDS supplemented with protease inhibitors (Roche Diagnostics)), heated at 95 °C for 20 min and centrifuged at 10,000*g* for 10 min. Proteins in supernatants were quantified using the Pierce BCA Protein Assay Kit (Thermo Fisher Scientific). An equal amount of protein was mixed with Laemmli loading buffer (EcoTech Biotechnology). Proteins were then separated using 10% or 12% SDS-PAGE and then transferred from the gel to a nitrocellulose membrane. The antibodies used are as follows: monoclonal mouse antibody (HL5511, 1:2,000, GeneTex) for the viral nucleoprotein, a polyclonal rabbit antibody (NBP1-76611, 1:1,000, Bio-Techne) for ACE2 and a monoclonal rabbit antibody (ab32370, 1:1,000, Abcam) for BCL-XL. The detection was made by using the appropriate horseradish peroxidase-conjugated secondary antibody (1:2,500, Jackson ImmunoResearch). Antibodies were detected using chemoluminescence (Pierce), and the signals were quantified by applying the ‘gel quantification’ procedure in ImageJ software (version 1.1.0) (National Institutes of Health). To normalize, an antibody directed against β-tubulin (86298, 1:1,000, Cell Signaling Technology) was used. For p16 detection in HeLa cells, monoclonal rabbit anti-p16 (ab211542, 1:1,000, Abcam) was used. The antibody against β-actin was from Sigma-Aldrich (A5441, 1:1,000).

### Determination of host gene expression using quantitative RT–PCR

Gene expression in the lungs was analyzed by quantitative RT–PCR as described^33^. Specific primers are shown in Table 2. Relative mRNA levels were determined according to the 2^−ΔΔ*C*t^ method by comparing (1) the PCR cycle thresholds (*C*t) for the gene of interest and the housekeeping gene (Δ*C*t) and (2) the Δ*C*t values for the treated and control groups (ΔΔ*C*t). Data were normalized against expression of the *Actg1 g*ene and expressed the fold change over the mean gene expression level in mock-treated young hamsters.

### Histopathological assessments

Lung tissues (left lobe) were fixed in 4% PBS-buffered formaldehyde for 7 d, rinsed in PBS, transferred into a 70% ethanol solution and processed into paraffin-embedded tissue blocks. The histological processing and analysis was subcontracted to Sciempath Labo, and histopathologic scores were given by a board-certified pathologist. Tissue sections (3 µm thick) were stained with H&E reagent. Whole-mount tissues were scanned with a Nanozoomer (Hamamatsu Photonics), and morphological changes were assessed by using a semi-quantitative dual histopathology score adapted from refs. ^33,57,82^. To evaluate pulmonary fibrosis, the Sirius Red-stained areas on scanned sections were measured with a computer-assisted, automated, whole-section histomorphometric image analysis technique (Visiopharm). Virtual whole sections were observed at a magnification of ×20 (corresponding to 0.46 μm per pixel). An algorithm for Sirius Red morphometric measurement on lung-stained sections was generated with the Bayesian linear segmentation tool in the Visiopharm software package and then refined by training on a subset of lung sections. Major histology section artifacts (such as large vascular and peribronchiolar structures and the alveolar lumen) were automatically delineated and removed from the area of interest. The Sirius Red-positive area (in mm^2^) was measured and expressed as a percentage of the total area of interest. The accuracy of the automated morphometric evaluation was checked on each individual image.

### Immunohistochemistry and immunofluorescence

Tissue sections (7 µm thick) were dried for 48 h at 42 °C. Slides were rehydrated with toluene (AnalaR NORMAPUR ACS, VWR) and decreasing concentrations of ethanol in water. The lung sections were stained with mouse monoclonal anti-p16 (sc-377412, 1:500, Santa Cruz Biotechnology) or rabbit polyclonal anti-SARS-CoV-2-spike glycoprotein (ab 272504, 1:5,000, Abcam) antibodies. For p16 and spike labeling (immunohistochemistry), the slides were blocked for endogenous peroxidase with 3% H_2_O_2_ and boiled for antigen retrieval in citrate buffer (0.1 M citric acid, 0.1 M dehydrated sodium citrate and milli-Q water (Millipore) pH 6). Sections were incubated with the appropriate secondary antibody from Vector Laboratories (goat anti-mouse IgG (H+L), biotinylated BA-9200-1.5 or goat anti-rabbit IgG (H+L), BA-1000-1.5, 1:200), washed and incubated with the VECTASTAIN Elite ABC Peroxidase Standard Kit (Vector Laboratories). Slides were washed three times in PBS, and the chromogen 3,3′-diaminobenzidine (DAB) from the Peroxidase Substrate Kit (SK-4100, Vector Laboratories) was added to each slide. The slides were counterstained with Mayer’s Hemalun (Merck). Lastly, the slides were mounted with glycerin mounting medium (glycergel mounting medium c0563, Dako). Images were acquired using an Axio Scan.Z1 slide scanner, ZEN (Blue edition) 2012 software (Carl Zeiss), a Leica DM3000 LED microscope and a FLEXACAM C1 camera. For p16, ACE2 and viral nucleoprotein labeling (immunofluorescence), the following antibodies were used: p16 (sc-377412, 1:50, Santa Cruz Biotechnology), ACE2 (NBP1-76611, 1:50, Bio-Techne) and SARS-CoV nucleocapsid (NB100-56576, 1:50, Bio-Techne). The rehydrated tissue sections were first treated with antigen unmasking solution (sodium citrate buffer pH 6 or Tris EDTA buffer pH 9). Then, sections were rinsed and blocked for 3 h at room temperature in blocking solution (PBS containing 5% BSA and 0.3% Triton X-100). Sections were incubated overnight at 4 °C with primary antibodies diluted in blocking solution. Sections were then washed and incubated at room temperature for 1 h with Alexa Fluor-conjugated secondary antibodies (A-11037, 1:500, Invitrogen) in blocking solution. For p16, a VectaFluorExcel Amplified Anti-Mouse IgG, DyLight 488 Antibody Kit (DK-2488, Vector Laboratories) was used according to the recommendations. Sections were stained with 4′,6-diamidino-2-phenylindole (DAPI) (Sigma-Aldrich) for 10 min, and coverslips were then mounted on slides using a fluorescence mounting medium (Agilent Technologies). Mounted slides were stored in the dark and at 4 °C until image acquisition. Immunofluorescence quantification was performed with ZEN 3.2 software and the Image Analysis module (Carl Zeiss). The intensity of the labeling was normalized by DAPI count. For each group, 2–3 whole lung sections from at least three different animals were quantified.

### Transcriptomic analyses

The hamster lung transcriptome (young versus aged) was analyzed with custom-designed hamster gene expression microarrays (4 × 44,000 v2, Agilent Technologies) and the one-color gene expression Agilent workflow. In brief, the microarrays were designed with the eArray server (Agilent Technologies) by starting from the *Mesocricetus auratus* gene annotation MesAur1.0 provided by Ensembl (Agilent AMAMID no. 086414). The custom microarrays were hybridized with 1,650 ng of Cy3-labeled cRNAs purified on RNeasy Mini-Spin Columns (Qiagen). Starting from 100 ng of total RNA, cRNAs were synthesized and labeled with Cy3 dye using the one-color Low Input Quick Amp Labeling Kit (Agilent Technologies). After hybridization for 17 h at 65 °C, the microarrays were washed and scanned with a G2565CA Agilent DNA microarray scanner. Fluorescence signals were extracted and normalized with Feature Extraction software (version 10.5.1.1, Agilent Technologies) and transferred to GeneSpring GX 12.6 software (Agilent Technologies) for processing and data mining. Expression data were normalized by applying the 75th percentile method in GeneSpring. To remove probes with a raw signal below 10 in all the conditions tested, the microarray probes were filtered with an Agilent flag filter. Samples from at least three independent hamsters in each group were analyzed, and differentially expressed genes were identified in a volcano plot with fold change cutoffs >1.5 or <1.5 and a moderated *t*-test *P* < 0.01 after Benjamani–Hochberg correction. The data were visualized by hierarchical clustering with the Euclidian metric and complete linkage. A GSEA was performed by using the pre-ranked routine and the default parameters in GSEA software (version 2.0.13). All gene set files for this analysis were obtained from the GSEA website (https://www.broadinstitute.org/gsea/). The CellAge database (https://genomics.senescence.info/cells/) was interrogated to identify genes related to cell senescence in aged hamsters. To study the effect of ABT-263 on gene expression in aged lungs, RNA sequencing was performed. In brief, RNA sequencing libraries were generated using the NEBNext Ultra II Directional RNA Library Prep Kit for Illumina (New England Biolabs (NEB)) with a poly(A) enrichment method following NEB’s recommendations. The libraries were sequenced on an Illumina NovaSeq platform (paired-end, 150 bp). The sequencing reads of each sample were trimmed and quality filtered using Trimmomatic (version 0.39) with the following options: ILLUMINACLIP:3:30:10 and MAXINFO:0.5. Cleaned reads were then processed using Salmon (version 1.9.0) with default parameters and the reference genome of *Mesocricetus auratus* (MesAur1.0 (GCA_000349665.1)). Gene annotations were computed using tximport (version 1.28.0). DESeq2 (version 1.12.3) was used to analyze the differential gene expression between the experimental groups. Some genes with a fold change > 1.5 (*P* < 0.01, moderated *t*-test after Benjamani–Hochberg correction) are represented in the hierarchical heat maps shown in Figs. 1c,d and 3g. Heat maps and clustering were generated from differential expression with Phantasus software (version 1.19.3). The hierarchical clustering was performed from the rows according to the matrix value metric (for a pre-computed similarity matrix) with a full linking method.

### Mass spectrometry analysis of the proteome and analysis of proteomics data

Spectral counting proteomic (serum) and tandem mass tag (TMT)-based proteomic (whole lung extract) were performed as follows. In brief, proteins (10 μg and 30 μg, respectively) were loaded on SDS–PAGE gels with gel slice trypsin digestion for each sample. For TMT-based proteomics (quadruplicates), peptides were labeled with TMT reagents (Thermo Fisher Scientific) according to the manufacturer’s instructions, and the different samples were mixed. Extracted peptides were fractionated with three acetonitrile increments (7.5%, 12.5% and 50%) for the spectral counting proteomic or eight acetonitrile increments for TMT-based proteomics in 0.1% triethylamine on a High pH Reversed-Phase Peptide Fractionation Kit (Thermo Fisher Scientific). Eluates were dried with a vacuum centrifuge and resolved in 0.1% formic acid. Peptides were separated by an UltiMate 3000 RSLCnano System and analyzed using Q Exactive instruments as previously described^83^. The raw nano liquid chromatography coupled to tandem mass spectrometry (LC–MS/MS) data were converted into an *.mgf peak list format, using Proteome Discoverer 1.4 (Thermo Fisher Scientific). MS/MS data were analyzed using the Mascot search engine (version 2.4.0, Matrix Science) installed on a local server. With a mass measurement tolerance of 10 ppm for precursors and 0.02 Da for fragment ions, we searched a composite target-decoy database (32,348 × 2 total entries) built from the UniProt *Mesocricetus auratus* dataset (taxonomy 10036, December 2021, 32,230 entries) fused with the sequences of recombinant trypsin and a standard list of contaminants (118 entries). Cysteine carbamidomethylation, methionine oxidation, protein N-terminal acetylation, cysteine propionamidation and TMT 6-plex (N-term and K) were searched for as variable modifications. Up to one missed trypsin cleavage was allowed. The identification results were imported into ProLine software (version 2.0) for validation. Peptide spectrum matches taller than nine residues and ion scores higher than 10 were retained. The false discovery rate (FDR) was then optimized to be below 1% at the protein level using the Mascot Modified MudPIT score. Spectral counting analyses were performed with ProLine 2.0. For normalization of TMT channels, the function ‘normalize to peptide amount’ was selected in Proteome Discoverer 1.4. Values correspond to the numbers of spectra per protein (spectral counting proteomic) and to the ratio of the abundance of the protein in a given sample divided by the abundance of the same protein in the pooled samples (TMT-based proteomic). Some proteins with a fold change >1.2 (serum) or >2 (lungs) (*P* < 0.2 and *P* < 0.05, respectively, two-tailed Mann–Whitney) are represented in the hierarchical heat maps shown in Fig. 5b and Fig. 5e, respectively.

### Statistical analysis and reproducibility

All experiments were performed at least two times except for Fig. 1a–d. For hamster experiments, 3–10 hamsters were analyzed per experiment. No power analyses were used to predetermine sample sizes, but our sample sizes were similar or superior to those reported in the previous publications^45,48^. Data distribution was assumed to be normal, but this was not formally tested. Data collection and analysis were not performed blinded to the conditions of the experiments. All statistical analyses were performed using GraphPad Prism version 9.2.0 software. Significance of body weight loss or regain (area under the curve) was calculated using the Wilcoxon matched-pairs signed-rank test. A two-tailed Mann–Whitney *U-*test was used to compare two groups, unless otherwise stated. Comparisons of more than two groups with each other were analyzed with the one-way ANOVA Kruskal–Wallis test (non-parametric), followed by Dunn’s post test. All data are expressed as the mean ± s.d.

### Reporting Summary

Further information on research design is available in the Nature Portfolio Reporting Summary linked to this article.

## Supplementary information

Reporting Summary

## Data Availability

Transcriptomic and proteomic raw data that support the findings of this study have been deposited in the Gene Expression Omnibus (GEO) and the Proteomics Identifications (PRIDE) database, respectively, with accession numbers GSE230301 (Fig. 1), GSE231673 (Fig. 3g), PXD041777 (Fig. 5b) and PXD041973 (Fig. 5e and Extended Data Fig. 7c) (http://www.ebi.ac.uk/pride).

## Source data

Source Data Fig. 2: Unprocessed western blots
Source Data Fig. 4: Unprocessed western blots
Source Data Extended Data 1: Unprocessed western blots
Source Data figures and extended data: Statistical Source Data

## Code availability

Codes and all other data supporting the findings of this study, including raw data underlying figures, are available from the corresponding author upon reasonable request.

## References

[1] Rea IM, Alexander HD (2022). Triple jeopardy in ageing: COVID-19, co-morbidities and inflamm-ageing. Ageing Res. Rev..

[2] Ruan Q, Yang K, Wang W, Jiang L, Song J (2020). Clinical predictors of mortality due to COVID-19 based on an analysis of data of 150 patients from Wuhan, China. Intensive Care Med..

[3] Williamson EJ (2020). Factors associated with COVID-19-related death using OpenSAFELY. Nature.

[4] O’Driscoll M (2021). Age-specific mortality and immunity patterns of SARS-CoV-2. Nature.

[5] Bartleson JM (2021). SARS-CoV-2, COVID-19 and the aging immune system. Nat. Aging.

[6] Boe DM, Boule LA, Kovacs EJ (2017). Innate immune responses in the ageing lung. Clin. Exp. Immunol..

[7] Schneider JL (2021). The aging lung: physiology, disease, and immunity. Cell.

[8] Yanagi S (2017). The impacts of cellular senescence in elderly pneumonia and in age-related lung diseases that increase the risk of respiratory infections. Int. J. Mol. Sci..

[9] Burton DGA, Stolzing A (2018). Cellular senescence: immunosurveillance and future immunotherapy. Ageing Res. Rev..

[10] He S, Sharpless NE (2017). Senescence in health and disease. Cell.

[11] Di Micco R, Krizhanovsky V, Baker D, d’Adda di Fagagna F (2021). Cellular senescence in ageing: from mechanisms to therapeutic opportunities. Nat. Rev. Mol. Cell Biol..

[12] Schmitt, C. A. et al. COVID-19 and cellular senescence. *Nat. Rev. Immunol.* **23**, 251–263 (2023). 10.1038/s41577-022-00785-2 PMC9533263 36198912

[13] Gorgoulis V (2019). Cellular senescence: defining a path forward. Cell.

[14] Barnes PJ, Baker J, Donnelly LE (2019). Cellular senescence as a mechanism and target in chronic lung diseases. Am. J. Respir. Crit. Care Med..

[15] Parikh P (2019). Cellular senescence in the lung across the age spectrum. Am. J. Physiol. Lung Cell. Mol. Physiol..

[16] Kirkland JL, Tchkonia T (2017). Cellular senescence: a translational perspective. EBioMedicine.

[17] Muñoz-Espín D, Serrano M (2014). Cellular senescence: from physiology to pathology. Nat. Rev. Mol. Cell Biol..

[18] Krizhanovsky V (2008). Senescence of activated stellate cells limits liver fibrosis. . Cell.

[19] Demaria M (2014). An essential role for senescent cells in optimal wound healing through secretion of PDGF-AA. Dev. Cell.

[20] Childs BG (2017). Senescent cells: an emerging target for diseases of ageing. Nat. Rev. Drug Discov..

[21] Baker DJ (2016). Naturally occurring p16^Ink4a^-positive cells shorten healthy lifespan. Nature.

[22] Chang J (2016). Clearance of senescent cells by ABT263 rejuvenates aged hematopoietic stem cells in mice. Nat. Med..

[23] Prata LGPL, Ovsyannikova IG, Tchkonia T, Kirkland JL (2018). Senescent cell clearance by the immune system: emerging therapeutic opportunities. Semin. Immunol..

[24] Tchkonia T, Zhu Y, van Deursen J, Campisi J, Kirkland JL (2013). Cellular senescence and the senescent secretory phenotype: therapeutic opportunities. J. Clin. Invest..

[25] Baker DJ (2011). Clearance of p16^Ink4a^-positive senescent cells delays ageing-associated disorders. Nature.

[26] Zhu Y (2016). Identification of a novel senolytic agent, navitoclax, targeting the Bcl-2 family of anti-apoptotic factors. Aging Cell.

[27] Sargiacomo C, Sotgia F, Lisanti MP (2020). COVID-19 and chronological aging: senolytics and other anti-aging drugs for the treatment or prevention of corona virus infection?. Aging (Albany NY).

[28] Wissler Gerdes EO (2022). Role of senescence in the chronic health consequences of COVID-19. Transl. Res..

[29] Kohli J, Veenstra I, Demaria M (2021). The struggle of a good friend getting old: cellular senescence in viral responses and therapy. EMBO Rep..

[30] Nehme J, Borghesan M, Mackedenski S, Bird TG, Demaria M (2020). Cellular senescence as a potential mediator of COVID-19 severity in the elderly. Aging Cell.

[31] Camell CD (2021). Senolytics reduce coronavirus-related mortality in old mice. Science.

[32] Weiss, S. R. & Leibowitz, J. L. Coronavirus pathogenesis. In *Advances in Virus Research* Vol. 81, 85–164 10.1016/B978-0-12-385885-6.00009-2 (Elsevier, 2011). 10.1016/B978-0-12-385885-6.00009-2 PMC7149603 22094080

[33] Sencio V (2022). Alteration of the gut microbiota following SARS-CoV-2 infection correlates with disease severity in hamsters. Gut Microbes.

[34] Sia SF (2020). Pathogenesis and transmission of SARS-CoV-2 in golden hamsters. Nature.

[35] Farr JN (2017). Targeting cellular senescence prevents age-related bone loss in mice. Nat. Med..

[36] Bussian TJ (2018). Clearance of senescent glial cells prevents tau-dependent pathology and cognitive decline. Nature.

[37] Piechota M (2016). Is senescence-associated β-galactosidase a marker of neuronal senescence?. Oncotarget.

[38] Dimri GP (1995). A biomarker that identifies senescent human cells in culture and in aging skin in vivo. Proc. Natl Acad. Sci. USA.

[39] Debacq-Chainiaux F, Erusalimsky JD, Campisi J, Toussaint O (2009). Protocols to detect senescence-associated beta-galactosidase (SA-βgal) activity, a biomarker of senescent cells in culture and in vivo. Nat. Protoc..

[40] Cai Y (2020). Elimination of senescent cells by β-galactosidase-targeted prodrug attenuates inflammation and restores physical function in aged mice. Cell Res..

[41] Selvaraj P (2021). SARS-CoV-2 infection induces protective immunity and limits transmission in Syrian hamsters. Life Sci. Alliance.

[42] Bogard G (2023). SARS-CoV-2 infection induces persistent adipose tissue damage in aged golden Syrian hamsters. Cell Death Dis..

[43] Chow RD, Majety M, Chen S (2021). The aging transcriptome and cellular landscape of the human lung in relation to SARS-CoV-2. Nat. Commun..

[44] Sepe S (2022). DNA damage response at telomeres boosts the transcription of SARS‐CoV‐2 receptor ACE2 during aging. EMBO Rep..

[45] Lee S (2021). Virus-induced senescence is a driver and therapeutic target in COVID-19. Nature.

[46] Lipskaia L (2022). Evidence that SARS-CoV-2 induces lung cell senescence: potential impact on COVID-19 lung disease. Am. J. Respir. Cell Mol. Biol..

[47] Wang S (2021). A single-cell transcriptomic landscape of the lungs of patients with COVID-19. Nat. Cell Biol..

[48] Tsuji S (2022). SARS-CoV-2 infection triggers paracrine senescence and leads to a sustained senescence-associated inflammatory response. Nat Aging.

[49] Evangelou, K. et al. Pulmonary infection by SARS-CoV-2 induces senescence accompanied by an inflammatory phenotype in severe COVID-19: possible implications for viral mutagenesis. *Eur. Respir. J.* **60**, 2102951 (2022). 10.1183/13993003.02951-2021 PMC8796696 35086840

[50] Seoane R, Vidal S, Bouzaher YH, El Motiam A, Rivas C (2020). The interaction of viruses with the cellular senescence response. Biology.

[51] Iba T, Levy JH, Levi M, Thachil J (2020). Coagulopathy in COVID-19. J. Thromb. Haemost..

[52] Al-Samkari H (2020). COVID-19 and coagulation: bleeding and thrombotic manifestations of SARS-CoV-2 infection. Blood.

[53] Ottenheijm CAC (2006). Activation of the ubiquitin–proteasome pathway in the diaphragm in chronic obstructive pulmonary disease. Am. J. Respir. Crit. Care Med..

[54] Roque W, Summer R, Romero F (2019). Fine-tuning the ubiquitin–proteasome system to treat pulmonary fibrosis. Connective Tissue Res..

[55] Woodside DG, Vanderslice P (2008). Cell adhesion antagonists: therapeutic potential in asthma and chronic obstructive pulmonary disease. BioDrugs.

[56] Slack RJ, Macdonald SJF, Roper JA, Jenkins RG, Hatley RJD (2022). Emerging therapeutic opportunities for integrin inhibitors. Nat. Rev. Drug Discov..

[57] Imai M (2020). Syrian hamsters as a small animal model for SARS-CoV-2 infection and countermeasure development. Proc. Natl Acad. Sci. USA.

[58] Osterrieder N (2020). Age-dependent progression of SARS-CoV-2 infection in Syrian hamsters. Viruses.

[59] Griffin BD (2021). Host parameters and mode of infection influence outcome in SARS-CoV-2-infected hamsters. iScience.

[60] Oishi K, Horiuchi S, Frere J, Schwartz RE, tenOever BR (2022). A diminished immune response underlies age-related SARS-CoV-2 pathologies. Cell Rep..

[61] Dinnon KH (2020). A mouse-adapted model of SARS-CoV-2 to test COVID-19 countermeasures. Nature.

[62] Sun S-H (2020). A mouse model of SARS-CoV-2 infection and pathogenesis. Cell Host Microbe.

[63] Beer J (2022). Impaired immune response drives age-dependent severity of COVID-19. J. Exp. Med..

[64] Meyer K, Patra T, Vijayamahantesh, Ray R (2021). SARS-CoV-2 spike protein induces paracrine senescence and leukocyte adhesion in endothelial cells. J. Virol..

[65] Kelley WJ, Zemans RL, Goldstein DR (2020). Cellular senescence: friend or foe to respiratory viral infections?. Eur. Respir. J..

[66] AbuBakar S, Shu M-H, Johari J, Wong P-F (2014). Senescence affects endothelial cells susceptibility to dengue virus infection. Int. J. Med. Sci..

[67] Baz-Martínez M (2016). Cell senescence is an antiviral defense mechanism. Sci. Rep..

[68] Kim J-A, Seong R-K, Shin OS (2016). Enhanced viral replication by cellular replicative senescence. Immune Netw..

[69] Hsieh T-H (2020). Senescence in monocytes facilitates dengue virus infection by increasing infectivity. Front. Cell. Infect. Microbiol..

[70] Maremanda KP, Sundar IK, Li D, Rahman I (2020). Age-dependent assessment of genes involved in cellular senescence, telomere, and mitochondrial pathways in human lung tissue of smokers, COPD, and IPF: associations with SARS-CoV-2 COVID-19 ACE2-TMPRSS2-Furin-DPP4 axis. Front. Pharmacol..

[71] de Moraes D (2021). Prediction of SARS-CoV interaction with host proteins during lung aging reveals a potential role for TRIB3 in COVID-19. Aging Dis..

[72] Ma S (2021). Single-cell transcriptomic atlas of primate cardiopulmonary aging. Cell Res..

[73] Bellini L (2020). Endoplasmic reticulum stress mediates resistance to BCL-2 inhibitor in uveal melanoma cells. Cell Death Discov..

[74] Lehmann M (2020). Chronic WNT/β-catenin signaling induces cellular senescence in lung epithelial cells. Cell Signal..

[75] Frere JJ (2022). SARS-CoV-2 infection in hamsters and humans results in lasting and unique systemic perturbations post recovery. Sci. Transl. Med..

[76] Hickson LJ (2019). Senolytics decrease senescent cells in humans: preliminary report from a clinical trial of dasatinib plus quercetin in individuals with diabetic kidney disease. EBioMedicine.

[77] Justice JN (2019). Senolytics in idiopathic pulmonary fibrosis: results from a first-in-human, open-label, pilot study. EBioMedicine.

[78] Verdoorn BP (2021). Fisetin for COVID-19 in skilled nursing facilities: senolytic trials in the COVID era. J. Am. Geriatr. Soc..

[79] Di Pierro F (2021). Possible therapeutic effects of adjuvant quercetin supplementation against early-stage COVID-19 infection: a prospective, randomized, controlled, and open-label study. Int. J. Gen. Med..

[80] Di Pierro F (2021). Potential clinical benefits of quercetin in the early stage of COVID-19: results of a second, pilot, randomized, controlled and open-label clinical trial. Int. J. Gen. Med..

[81] Shohan M (2022). The therapeutic efficacy of quercetin in combination with antiviral drugs in hospitalized COVID-19 patients: a randomized controlled trial. Eur. J. Pharmacol..

[82] Meyerholz DK, Beck AP (2020). Histopathologic evaluation and scoring of viral lung infection. Methods Mol. Biol..

[83] Paiva I (2022). Caffeine intake exerts dual genome-wide effects on hippocampal metabolism and learning-dependent transcription. J. Clin. Invest..

