# Supplementary material for: Removal of senescent cells reduces the viral load and attenuates pulmonary and systemic inflammation in SARS-CoV-2-infected, aged hamsters
Source: Nat Aging. 2023 Jul 6;3(7):829–45. doi: 10.1038/s43587-023-00442-w (PMC10353934; doi:10.1038/s43587-023-00442-w)
Supplement: Supplementary file 1 — Reporting Summary [file 43587_2023_442_MOESM1_ESM.pdf]

nature portfolio

Corresponding author(s): François TROTTEIN

Last updated by author(s): 2023/05/05

Reporting Summary

Nature Portfolio wishes to improve the reproducibility of the work that we publish. This form provides structure for consistency and transparency in reporting. For further information on Nature Portfolio policies, see our Editorial Policies and the Editorial Policy Checklist.

Statistics

For all statistical analyses, confirm that the following items are present in the figure legend, table legend, main text, or Methods section.

n/a	Confirmed	
☐	☒	The exact sample size (n) for each experimental group/condition, given as a discrete number and unit of measurement
☐	☒	A statement on whether measurements were taken from distinct samples or whether the same sample was measured repeatedly
☐	☒	The statistical test(s) used AND whether they are one- or two-sided<br>*Only common tests should be described solely by name; describe more complex techniques in the Methods section.*
☒	☐	A description of all covariates tested
☒	☐	A description of any assumptions or corrections, such as tests of normality and adjustment for multiple comparisons
☐	☒	A full description of the statistical parameters including central tendency (e.g. means) or other basic estimates (e.g. regression coefficient) AND variation (e.g. standard deviation) or associated estimates of uncertainty (e.g. confidence intervals)
☐	☒	For null hypothesis testing, the test statistic (e.g. F, t, r) with confidence intervals, effect sizes, degrees of freedom and P value noted<br>*Give P values as exact values whenever suitable.*
☒	☐	For Bayesian analysis, information on the choice of priors and Markov chain Monte Carlo settings
☒	☐	For hierarchical and complex designs, identification of the appropriate level for tests and full reporting of outcomes
☒	☐	Estimates of effect sizes (e.g. Cohen's d, Pearson's r), indicating how they were calculated

Our web collection on statistics for biologists contains articles on many of the points above.

Software and code

Policy information about availability of computer code

Data collection	Microscopic data: AxioImager 4 (Zeiss), DM3000 microscope (Leica), Flexacam C1 (Leica), EVOS M5000 Imaging System (Thermo Fisher Scientific)<br>Quantitative real-time PCR: QuantStudio 12K Flex Real-Time PCR System (Thermo Fisher Scientific)<br>Immunoblotting data: ChemiDoc MP System (Bio-Rad)<br>Histological data: Nanozoomer (Hamamatsu photonics) and Axio Scan.Z1 slide scanner (Zeiss)<br>Transcriptomic data: G2565CA DNA microarray scanner (Agilent Technology) , NovaSeq6000 (Illumina)<br>Mass spectrometry and proteomics data: Q-Exactive instruments (Thermo Fisher Scientific)
Data analysis	Immunoblotting: Image J software (version 1.1.0)<br>Immunofluorescence staining: ZEN 3.2 software and the Image Analysis module (version 3.2)<br>Histological: Visiopharm software package<br>Transcriptomic data (Agilent): Agilent feature extraction software (version 10.5.1.1) and transfer to Genespring GX 12.6 software, GSEA software (version 2.0.13)<br>Transcriptomic data (RNAseq): Trimmomatic (v0.39), Salmon (v1.9.0), tximport (v1.28.0), DESeq2 (v1.12.3)<br>Proteomic analysis: Mascot search engine (version 2.4.), Proline 2.0<br>Heatmap : Phantasus v1.19.3<br>Software statistical analysis: GraphPad Prism 9 version 9.2.0

For manuscripts utilizing custom algorithms or software that are central to the research but not yet described in published literature, software must be made available to editors and reviewers. We strongly encourage code deposition in a community repository (e.g. GitHub). See the Nature Portfolio guidelines for submitting code & software for further information.

Data

Policy information about availability of data

All manuscripts must include a data availability statement. This statement should provide the following information, where applicable:

- Accession codes, unique identifiers, or web links for publicly available datasets
- A description of any restrictions on data availability
- For clinical datasets or third party data, please ensure that the statement adheres to our policy

Transcriptomic and proteomic raw data that support the findings of this study have been deposited in Gene Expression Omnibus (GEO) and Proteomics IDEntifications database (PRIDE), respectively with the accession numbers GSE230301 (Figure 1) (https://www.ncbi.nlm.nih.gov/geo/query/acc.cgi?acc=GSE230301), GSE231673 (Figure 3G) (https://www.ncbi.nlm.nih.gov/geo/query/acc.cgi?acc=GSE231673), PXD041777 (Figure. 5B), and PXD041973 (Figure 5.E and extended Figure 7C) (http://www.ebi.ac.uk/pride).

Research involving human participants, their data, or biological material

Policy information about studies with human participants or human data. See also policy information about sex, gender (identity/presentation), and sexual orientation and race, ethnicity and racism.

Reporting on sex and gender	N/A
Reporting on race, ethnicity, or other socially relevant groupings	N/A
Population characteristics	N/A
Recruitment	N/A
Ethics oversight	N/A

Note that full information on the approval of the study protocol must also be provided in the manuscript.

Field-specific reporting

Please select the one below that is the best fit for your research. If you are not sure, read the appropriate sections before making your selection.

☒ Life sciences ☐ Behavioural & social sciences ☐ Ecological, evolutionary & environmental sciences

For a reference copy of the document with all sections, see nature.com/documents/nr-reporting-summary-flat.pdf

Life sciences study design

All studies must disclose on these points even when the disclosure is negative.

Sample size	The sample size used in this study was determined based on the expense of data collection, and the need to have statistical power.
Data exclusions	in Fig. 2F, one individual who died before day 22 were excluded. For the other experiments, no data was excluded from the analysis
Replication	All data presented were obtained from two to three independent experiments with similar outcomes. Data shown in Figure 1A-D have been obtained from a single experiment (n=3-4)
Randomization	For all experiments, animals and/or cell culture wells were randomly assigned to experimental groups.
Blinding	Data collection and analysis were not performed blind to the conditions of the experiments, because experiments were performed and analyzed by the same researchers. However, experiments and analysis were performed objective and unbiaised.

Reporting for specific materials, systems and methods

We require information from authors about some types of materials, experimental systems and methods used in many studies. Here, indicate whether each material, system or method listed is relevant to your study. If you are not sure if a list item applies to your research, read the appropriate section before selecting a response.

Materials & experimental systems

n/a	Involved in the study	
☐	☒	Antibodies
☐	☒	Eukaryotic cell lines
☒	☐	Palaeontology and archaeology
☐	☒	Animals and other organisms
☒	☐	Clinical data
☒	☐	Dual use research of concern
☒	☐	Plants

Methods

n/a	Involved in the study	
☒	☐	ChIP-seq
☒	☐	Flow cytometry
☒	☐	MRI-based neuroimaging

Antibodies

Antibodies used

SARS-CoV-2 spike protein (Abcam, 272504) (1:5000), SARS-CoV-2 Nucleocapsid protein (Bio-Techne, NB100-56576) (1 :50), SARS-CoV-2 Nucleocapsid protein (GeneTex, GTX635689, clone HL5511) (1:2000), p16INK4a (Santa Cruz, sc-377412, clone C-7) (1:500, 1:50 (immunofluorescence)), p16INK4a (Abcam, ab211542, clone EPR20418) (1:1000), ACE2 (Bio-Techne, NBP1-76611) (1:1000, 1:50 (immunofluorescence)), ACE2 (Abcam, ab32370) (1:1000), Bcl-xL (abcam, ab32370, clone E18) (1 :1000), beta-tubulin (Cell Signaling, 86298, clone D3U1W) (1:1000), beta- actin (Sigma, A5441, clone AC-15) (1:1000).

Biotinylated goat anti-rabbit IgG (Vector Laboratories, BA-1000-1.5) (1:200), Biotinylated goat anti-mouse IgG (Vector Laboratories, BA-9200-1.5) (1:200), Goat anti-rabbit IgG Alexa Fluor 594 (Invitrogen, A-11037) (1:500), Goat anti-mouse IgG Alexa Fluor 488 (Invitrogen, A-11029) (1:500), HRP-conjugated goat anti-rabbit IgG (Abcam, ab6721), HRP-conjugated goat anti-mouse IgG (Abcam, ab6789) (1:2500), VectaFluor Excel Amplified Anti-Mouse IgG, DyLight”" 488 Antibody Kit (Vector Laboratories, DK-2488),Goat anti Mouse HRP (Abcam, ab6789) and goat anti Rabbit HRP (Abcam, ab6721) (1:2500).

Validation

The two anti-p16 antibodies, namely Santa Cruz/sc377412 and Abcam/ab211542, used in this study label HeLa cells transfected with hamster p16, but not control cells (Extended data Fig. 1). Silencing p16 expression by means of siRNAs abrogates the binding.

All antibodies used in this study were commercially available antibodies and were validated by companies. Data sheet is available from the web links as described below.
SARS-CoV-2 spike protein: https://www.abcam.com/products/primary-antibodies/sars-cov-2-spike-glycoprotein-antibody-coronavirus-ab272504.html
SARS-CoV-2 Nucleocapsid protein: https://www.novusbio.com/products/sars-nucleocapsid-protein-antibody nb100-56576 https://www.genetex.com/Product/Detail/SARS-CoV-2-COVID-19-Nucleocapsid-antibody-HL5511/GTX635689
p16INK4a: https://www.scbt.com/fr/p/p15-p16-antibody-c-7
https://www.abcam.com/products/primary-antibodies/cdkn2ap16ink4a-antibody-epr20418-ab211542.html#lb ACE2: https://www.novusbio.com/products/ace-2-antibody nbp1-76611
Bcl-xL: https://www.abcam.com/products/primary-antibodies/bcl-xl-antibody-e18-ab32370.html
B-tubulin: https://www.cellsignal.com/products/primary-antibodies/b-tubulin-d3u1w-mouse-mab/86298
B-actin: https://www.sigmaaldrich.com/FR/fr/product/sigma/a5441?gclid=CjwKCAjw ihBhADEiwAXEazJhN4w-MPakobsQDueVPhxMpwnspLuOlz8wmS8Q6NhSKbiDLXNbQcuhoC6bsQAvD BwE&gclsrc=aw.ds
Goat anti-rabbit IgG, biotinylated: https://vectorlabs.com/products/antibodies/biotinylated-goat-anti-rabbit-igg Goat anti-mouse IgG, biotinylated: https://vectorlabs.com/products/antibodies/biotinylated-goat-anti-mouse-igg
Goat anti-rabbit IgG Alexa Fluor 594: https://www.thermofisher.com/antibody/product/Goat-anti-Rabbit-IgG-H-L-Highly-Cross-Adsorbed-Secondary-Antibody-Polyclonal/A-11037
Goat anti-mouse IgG Alexa Fluor 488: https://www.thermofisher.com/antibody/product/Goat-anti-Mouse-IgG-H-L-Highly-Cross-Adsorbed-Secondary-Antibody-Polyclonal/A-11029
VectaFluor Excel Amplified Anti-Mouse IgG, DyLight”" 488 Antibody Kit: https://vectorlabs.com/products/antibodies/vectafluor-excel- dylight-488-anti-mouse-igg-kit
HRP-conjugated goat anti-rabbit IgG: https://www.abcam.com/products/secondary-antibodies/goat-rabbit-igg-hl-hrp-ab6721.html
HRP-conjugated goat anti-mouse IgG: https://www.abcam.com/products/secondary-antibodies/goat-mouse-igg-hl-hrp-ab6789.html

Eukaryotic cell lines

Policy information about cell lines and Sex and Gender in Research

Cell line source(s)

HeLa cells were obtained from ATCC (CCL-2), VERO-E6 cells were obtained from ATCC (CRL-1586), VERO/TMPRSS2 cells were produced in our laboratory (https://doi.org/10.1371/journal.ppat.1010498)

Authentication

These cells were not authenticated by ourselves

Mycoplasma contamination

We confirmed that there were no Mycoplasma contamination in our cultures

Commonly misidentified lines
(See ICLAC register)

No commonly misdentified cell lines were used in this study

Animals and other research organisms

Policy information about studies involving animals; ARRIVE guidelines recommended for reporting animal research, and Sex and Gender in Research

Laboratory animals	2 month-old and 22 month-old Syrian golden hamsters were purchased from Janvier Laboratory. The animals were fed a standard rodent chow (SAFE, Augy France) and were given water ad libitum.
Wild animals	The study did not involve wild animals
Reporting on sex	Findings described in this study apply for male hamsters
Field-collected samples	No field collected samples were used in the study.
Ethics oversight	All experiments involving SARS-CoV-2 were performed within the biosafety level 3 facility of the Institut Pasteur de Lille. The protocols were validated by the local committee for the evaluation of the biological risks and complied with current national and institutional regulations and ethical guidelines (Institut Pasteur de Lille/B59-350009). The experimental protocols using animals were approved by the institutional ethical committee “Comite´ d’Ethique en Experimentation Animale (CEEA) 75, Nord Pas-de-Calais”. The animal study was authorized by the “Education, Research and and Innovation Ministry” under registration number APAFIS#25041-2020040917227851v3.

Note that full information on the approval of the study protocol must also be provided in the manuscript.
