# Supplementary figures and images for: Removal of senescent cells reduces the viral load and attenuates pulmonary and systemic inflammation in SARS-CoV-2-infected, aged hamsters
Source: Nat Aging. 2023 Jul 6;3(7):829–45. doi: 10.1038/s43587-023-00442-w (PMC10353934; doi:10.1038/s43587-023-00442-w)

# Figure 2E

1-6 : Young

7-17 : Aged

Same membrane

1 2 3 4 5 6 7 8 9 10 11 12 13 14

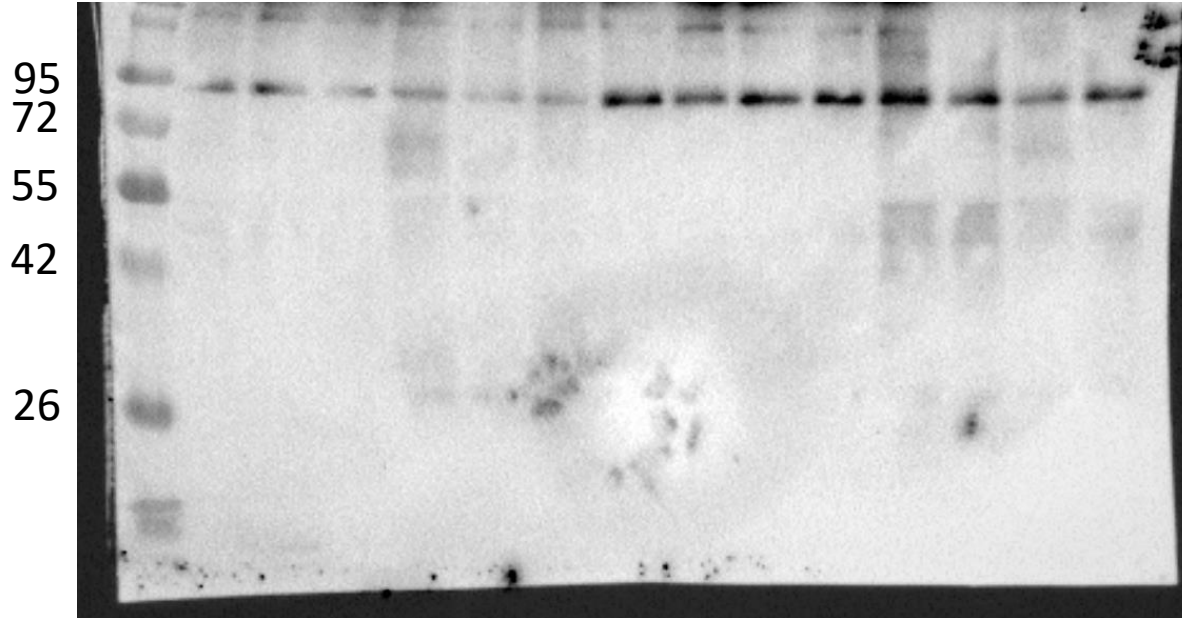

ACE2 (NBP1-76611)  
1/1000

1 2 3 4 5 6 7 8 9 10 11 12 13 14

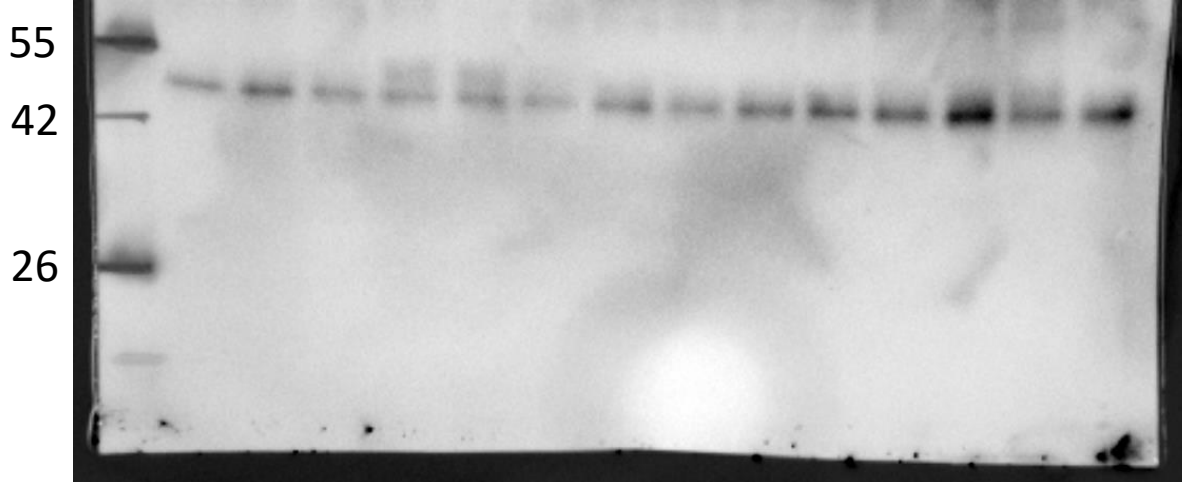

House keeping gene :  
 $\beta$ -tubulin (86298)  
1/1000

Supplement: Source Data Fig. 2: — Unprocessed western blots [file 43587_2023_442_MOESM2_ESM.pdf]
