# Supplementary material for: Removal of senescent cells reduces the viral load and attenuates pulmonary and systemic inflammation in SARS-CoV-2-infected, aged hamsters
Source: Nat Aging. 2023 Jul 6;3(7):829–45. doi: 10.1038/s43587-023-00442-w (PMC10353934; doi:10.1038/s43587-023-00442-w)
Supplement: Source Data Fig. 4: — Unprocessed western blots [file 43587_2023_442_MOESM3_ESM.pdf]

Figure 4E

1-5 : Vh   6-13 : ABT-263

Same membrane

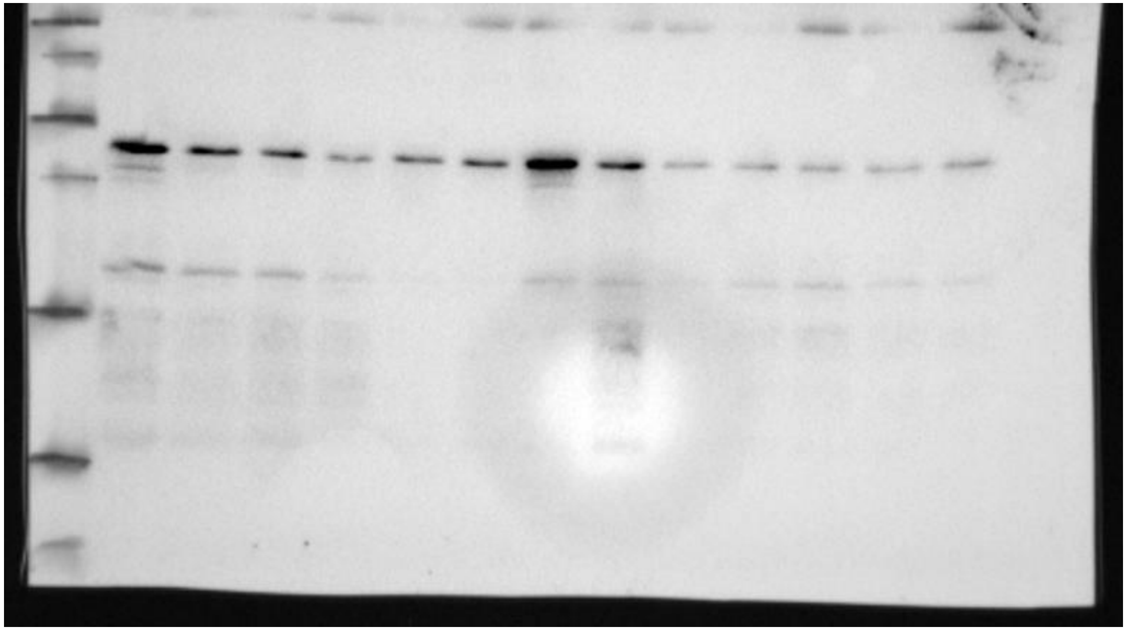

Nucleocapside SARS-CoV-2
(GTX65689)
1/2000

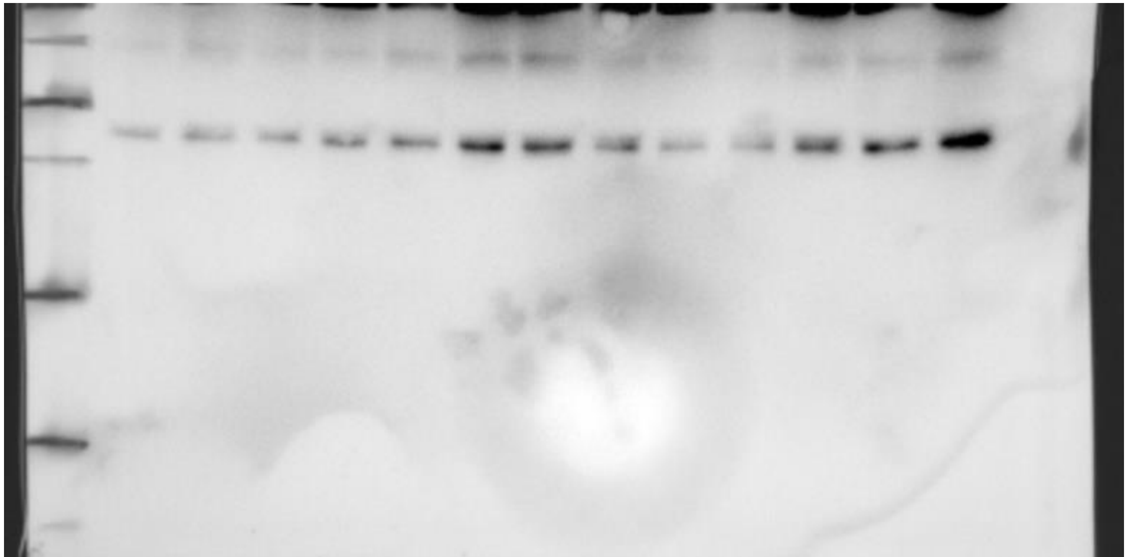

House keeping gene :
β-tubulin (86298)
1/1000

Same membrane

Figure 4F

1-3 : Vh 4-9 : ABT-263

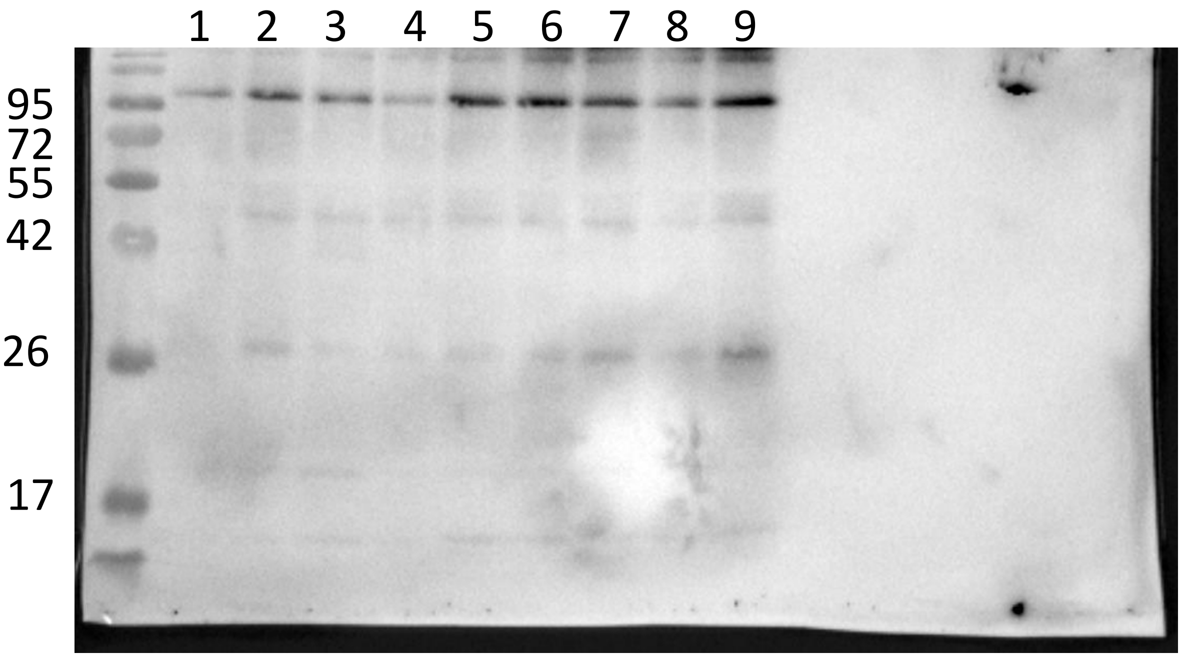

ACE2 (NBP1-76611)
1/1000

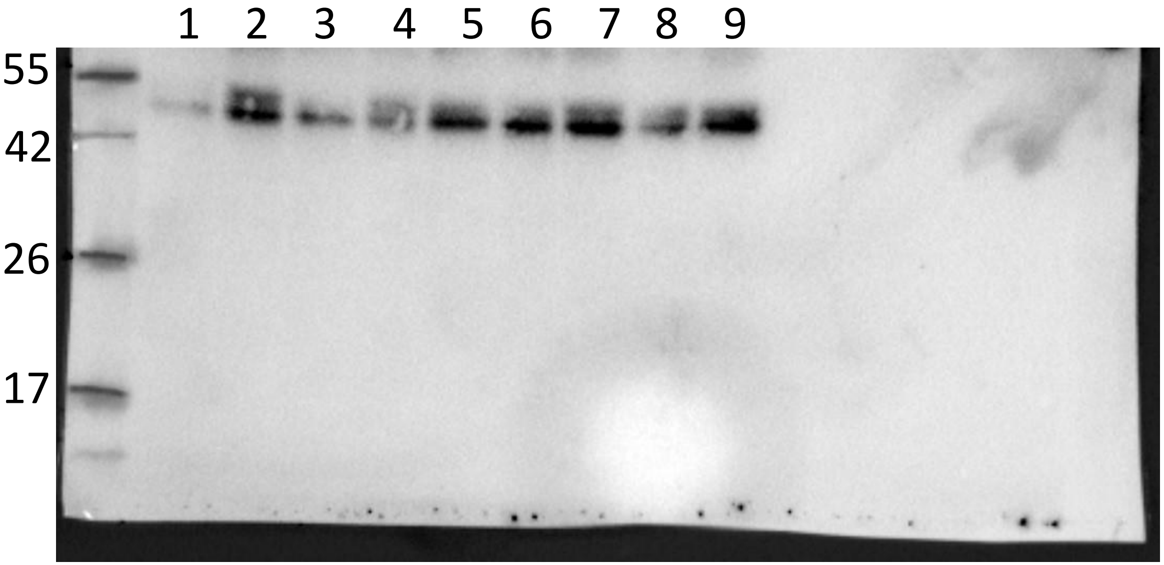

House keeping gene :
β-tubulin (86298)
1/1000
