# Supplementary material for: Removal of senescent cells reduces the viral load and attenuates pulmonary and systemic inflammation in SARS-CoV-2-infected, aged hamsters
Source: Nat Aging. 2023 Jul 6;3(7):829–45. doi: 10.1038/s43587-023-00442-w (PMC10353934; doi:10.1038/s43587-023-00442-w)
Supplement: Source Data Extended Data 1: — Unprocessed western blots [file 43587_2023_442_MOESM4_ESM.pdf]

Same membrane

Extended data Figure 1A

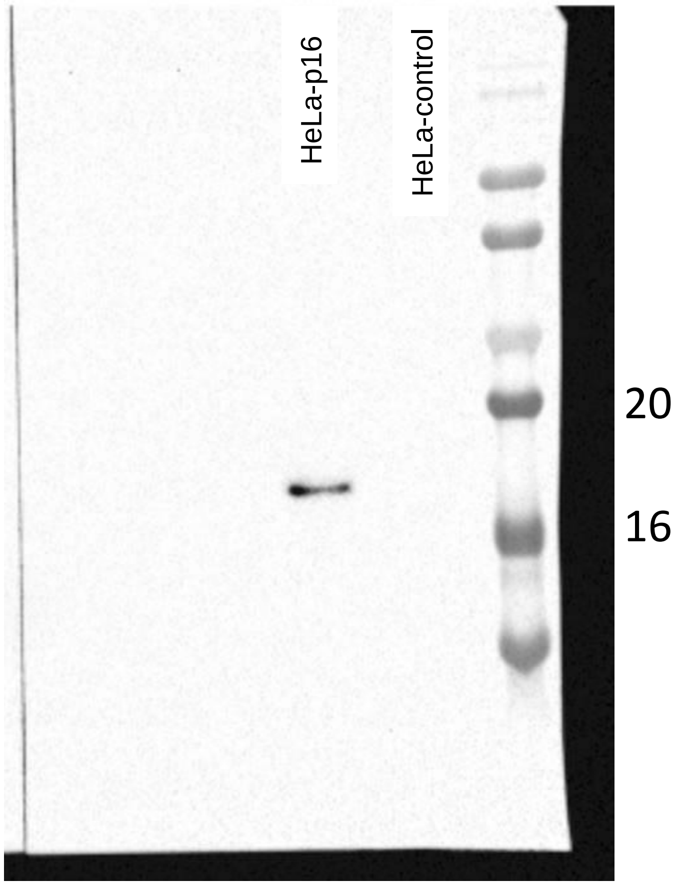

p16 (ab211542)
1/1000

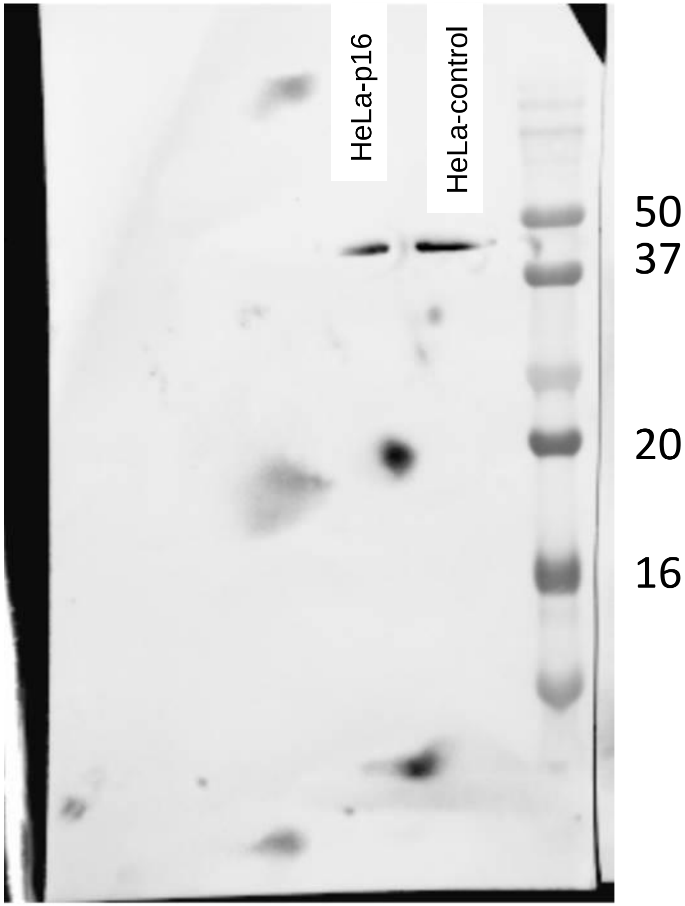

House keeping gene :
β-actin (A5441)
1/1000

Extended data Figure 1D

Same membrane

1-3 : Young 4-9 : Aged

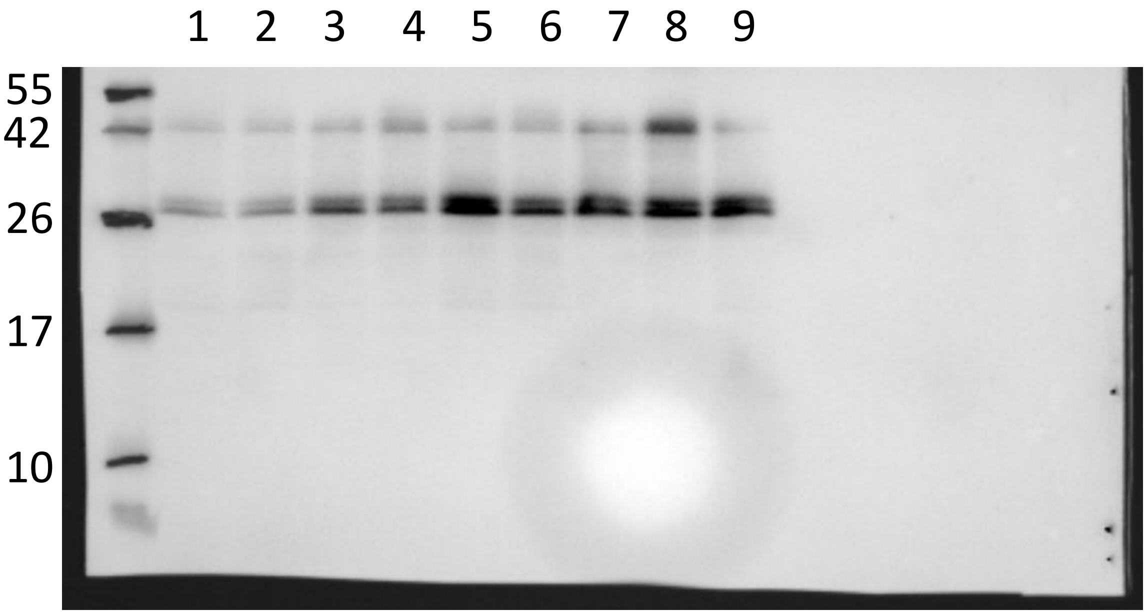

BCL-XL (ab32370)
1/100

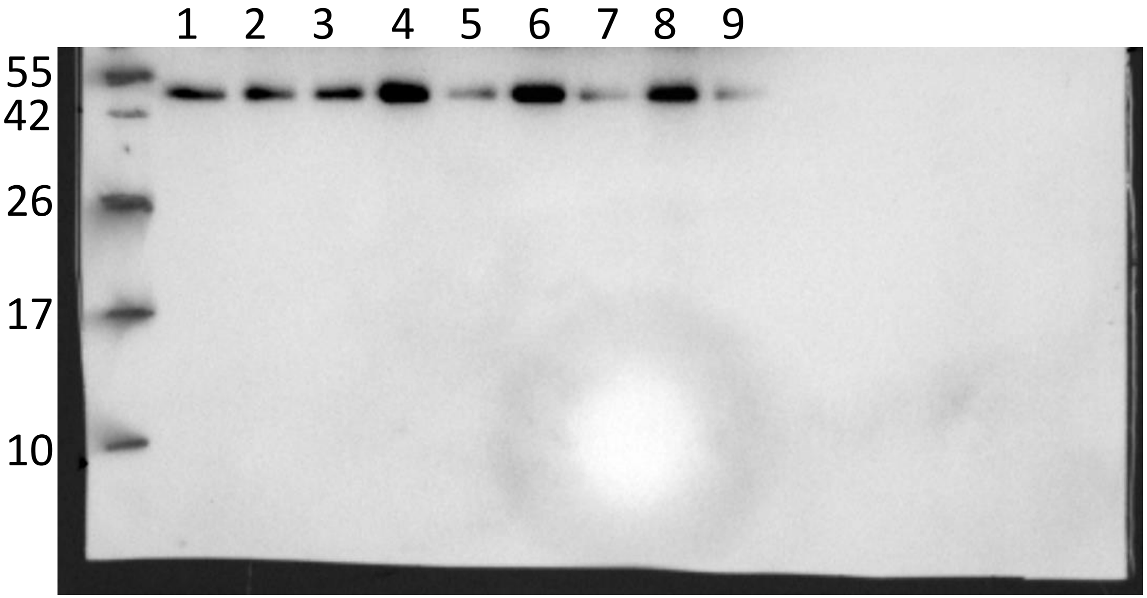

House keeping gene :
β-tubulin (86298)
1/1000
